# Genetically Encoded Tools for Research of Cell Signaling and Metabolism under Brain Hypoxia

**DOI:** 10.3390/antiox9060516

**Published:** 2020-06-11

**Authors:** Alexander I. Kostyuk, Aleksandra D. Kokova, Oleg V. Podgorny, Ilya V. Kelmanson, Elena S. Fetisova, Vsevolod V. Belousov, Dmitry S. Bilan

**Affiliations:** 1 Shemyakin-Ovchinnikov Institute of Bioorganic Chemistry, 117997 Moscow, Russia; alexander.kostyuk@inbox.ru (A.I.K.); ms.demidovich@inbox.ru (A.D.K.); olegpodgorny@inbox.ru (O.V.P.); ikelmanson@gmail.com (I.V.K.); fetisova.el.s@gmail.com (E.S.F.); vsevolod.belousov@gmail.com (V.V.B.); 2 Center for Precision Genome Editing and Genetic Technologies for Biomedicine, Pirogov Russian National Research Medical University, 117997 Moscow, Russia; 3 Koltzov Institute of Developmental Biology, 119334 Moscow, Russia; 4 Faculty of Biology, Lomonosov Moscow State University, 119992 Moscow, Russia; 5 Institute for Cardiovascular Physiology, Georg August University Göttingen, D-37073 Göttingen, Germany; 6 Federal Center for Cerebrovascular Pathology and Stroke, 117997 Moscow, Russia

**Keywords:** oxygen, hypoxia, hypoxia-inducible factor (HIF), genetically encoded biosensors, fluorescent proteins, luciferase

## Abstract

Hypoxia is characterized by low oxygen content in the tissues. The central nervous system (CNS) is highly vulnerable to a lack of oxygen. Prolonged hypoxia leads to the death of brain cells, which underlies the development of many pathological conditions. Despite the relevance of the topic, different approaches used to study the molecular mechanisms of hypoxia have many limitations. One promising lead is the use of various genetically encoded tools that allow for the observation of intracellular parameters in living systems. In the first part of this review, we provide the classification of oxygen/hypoxia reporters as well as describe other genetically encoded reporters for various metabolic and redox parameters that could be implemented in hypoxia studies. In the second part, we discuss the advantages and disadvantages of the primary hypoxia model systems and highlight inspiring examples of research in which these experimental settings were combined with genetically encoded reporters.

## 1. Introduction

The evolution of organic life on Earth was accompanied by dramatic changes in the environmental oxygen concentration. Thus, the emergence of photosynthetic organisms that utilize CO_2_ and light energy for their metabolism, with molecular oxygen as a byproduct, led to the pronounced accumulation of the latter in the atmosphere [1]. Because molecular oxygen is a potent oxidizing agent, cells were forced to adapt to the novel conditions by developing antioxidant systems to protect their molecular structures; however, it paved the way to generate energy via the highly effective process of aerobic respiration [1]. As a result, aerobic organisms became strongly dependent on the oxygen supply; the gradual increase in anatomical and morphological complexity, including the evolution of multicellular forms, required a resolution of the problem of oxygen accumulation and transport. In vertebrates, the architectural solution is represented by the cardiovascular system, which includes oxygen-binding molecules (substrate carriers), blood vessels (the delivery routes), and a heart (the pump). The need to measure oxygen availability led to the emergence of various biochemical systems that act as oxygenation sensors and help to maintain energy homeostasis that is crucial for survival in changing environments [2,3].

When oxygen concentrations become reduced, cells experience hypoxia. In some situations, hypoxia is a “physiological” condition, and it plays a role in metabolism and developmental regulation. In particular, the embryonic morphogenesis of some organs, such as the placenta, heart, and bones, proceeds in the presence of low oxygen concentrations that induce relevant cell signaling [4]. In the adult state, “physiological” hypoxia maintains undifferentiated states of hematopoietic, mesenchymal, and neural stem cells and protects their DNA from oxidative damage [5]. The process of wound healing also includes the development of “physiological” hypoxia [6,7]. In other situations, the lack of oxygen is an undesirable event; hence, “non-physiological” hypoxia emerges. Living organisms are capable of protecting themselves from this condition to some extent on different organizational levels. Thus, cells reduce energy-intensive processes such as protein translation and ion channel functioning; in addition, they switch their metabolism from aerobic respiration to anaerobic glycolysis [8]. The effects on the higher organization levels include fast physiological reactions such as systemic blood vessel relaxation [9] and pulmonary blood vessel contraction [10], as well as slow reactions that require cell proliferation such as blood vessel growth [11] and erythropoiesis [12]. However, in some cases, organisms fail to handle hypoxia in an adequate way, which subsequently leads to the development of various diseases. Pulmonary arterial hypertension, obstructive sleep apnea, pressure-overload heart failure, and peripheral and coronary artery diseases represent just some examples of pathological conditions in which hypoxia plays the key role [13]. A significant body of accumulating experimental evidence reveals the effects of low oxygen concentrations on tumor metabolism, which consist in the reprogramming of energy metabolism, as well as in the promoting cell immortalization, vascularization, metastasis, and invasion [13,14]. Finally, the investigation of brain hypoxia is an area of heightened interest because the brain and its cells, due to high metabolic activity, are exceptionally vulnerable to injury from oxygen deprivation. 

Brain hypoxia/ischemia research primarily aims to (i) understand the molecular and genetic mechanisms underlying the pathogenesis of hypoxic injury and the vulnerability of the brain to hypoxia; (ii) discover and test novel drugs; and (iii) explain hypoxic/ischemic pre- and post-conditioning phenomena. The primary models for studying hypoxia of the central nervous system (CNS) include the following: (i) primary cell cultures (mainly derived from the embryonic or early postnatal brain, retina, or spinal cord), neuronal cell cultures, or cerebral organoids derived from embryonic stem cell lines (ESCs) or induced pluripotent stem cell lines (iPSCs); (ii) brain slice systems (acute slices and organotypic slice culture) and retina explants; and (iii) model organisms (primarily rodents). 

Traditional methodologies used for the detection and evaluation of hypoxic damage and oxidative stress in the CNS include biochemical assays, morphological analysis, and electrophysiological recordings. Biochemical assays for any type of biological sample (cultured cells, live tissue slices, or model organisms) are generally limited to the estimation of the following: the glutathione system; oxidative stress markers such as nitric oxide and hydrogen peroxide; lipid peroxidation; lactate dehydrogenase activity; and levels of antioxidant enzymes such as superoxide dismutase, glutathione peroxidase, and glutathione reductase. Morphological assays, which are performed by using light and electron microscopy techniques, primarily evaluate the following: cell death; tissue remodeling; and the structural integrity of neuropil, synapses, or cellular components such as nuclei, mitochondria, and the plasma membrane after exposure to hypoxia or ischemia. Biochemical and morphological evaluations require fixation or destruction of biological samples and can be performed only as the final step of an experiment. Therefore, the major disadvantage of these types of evaluations is a low temporal and spatial resolution of the readout. For many years, electrophysiological recording was the only methodology that enabled the assessment of cell functioning in real time during the course of hypoxic exposure. However, electrophysiological recordings only allow for the measurement of a limited number of cell parameters with poor spatial resolution.

Progress in studying hypoxic/ischemic injury of the CNS only began when the following methods became available: powerful platforms for live imaging that were primarily reliant on optical methods (fluorescence, confocal and two-photon microscopy, and photoacoustic tomography [15]); nuclear technologies such as magnetic resonance imaging (MRI) and positron emission tomography (PET) [16,17], and a plethora of chemogenic and genetically encoded fluorescent indicators for probing a variety of biologically significant ions, signaling molecules, and metabolites, including those involved in redox regulation [18,19,20,21,22]. Although they are the principal tools used for clinical and diagnostic purposes, nuclear technologies provide insufficient spatiotemporal resolution of brain functioning at a cellular level. Therefore, optical imaging combined with the use of fluorescent indicators is a fundamental tool used in basic research; this method enables the noninvasive detection of various intracellular metabolites or signaling molecules in real time with single-cell or even subcellular resolution. Genetically encoded biosensors have many advantages over other types of fluorescent probes (reviewed in [20,21,23,24,25,26]) and have allowed unmatched opportunities for studying hypoxic/ischemic injury of the brain. Genetically encoded biosensors may be expressed in a spatiotemporally defined manner in specific cell types or subcellular compartments. This provides high flexibility of experimental design and enhances the resolution of cell analysis. They may be used for creating stable cell lines [27] or even transgenic animals [28,29,30,31]. This has enabled researchers to overcome the challenges associated with using a fluorescent probe in each individual experiment, thus simplifying experimental design and excluding additional procedures that may perturb the results. These stable cell lines and transgenic animals can be used as unique models for studying the pathogenesis of hypoxia or discovering and testing new drugs. Genetically encoded biosensors are compatible with super-resolution microscopy [32], which enables scientists to gain deep insights into the kinetics of biochemical reactions in the cell [33,34]. The live probing of cell signaling and metabolism with genetically encoded biosensors during the course of hypoxic exposure provides multiparametric readouts that are important for understanding key molecular events associated with tissue response, vulnerability, and the initiation of pathogenic processes. Surprisingly, despite their conspicuous utility for brain hypoxia research, genetically encoded biosensors are infrequently used in this field. 

The current paper establishes two main objectives: (i) to describe the palette of genetically encoded reporters that could be implemented in brain hypoxia research and (ii) to discuss existing research that has incorporated these methods. We, therefore, hope that it will encourage future researchers to enter this fruitful scientific field. 

## 2. Genetically Encoded Reporters for Hypoxia Investigation

In this section we describe genetically encoded reporters that could be implemented to investigate brain hypoxia. It includes two subsections: (i) genetically encoded reporters that are specific for oxygen/hypoxia and (ii) genetically encoded reporters for the cellular processes whose changes are crucial to the understanding of cellular metabolism in hypoxic conditions. 

### 2.1. Genetically Encoded Reporters for Oxygen Detection

The spatiotemporal changes of oxygen concentrations represent an independent aspect of interest in hypoxia research. The correlation between the time of hypoxia onset/termination or the degree of its severity with biochemical parameters and physiological outcomes is of great importance for understanding how different cells tolerate low oxygen concentration. This subsection is dedicated to genetic tools that enable the optical visualization of either oxygen concentration or hypoxia-induced cellular signaling. 

#### 2.1.1. Natural Oxygen-Sensing Platforms for the Development of Oxygen Reporters

**Figure 1:**
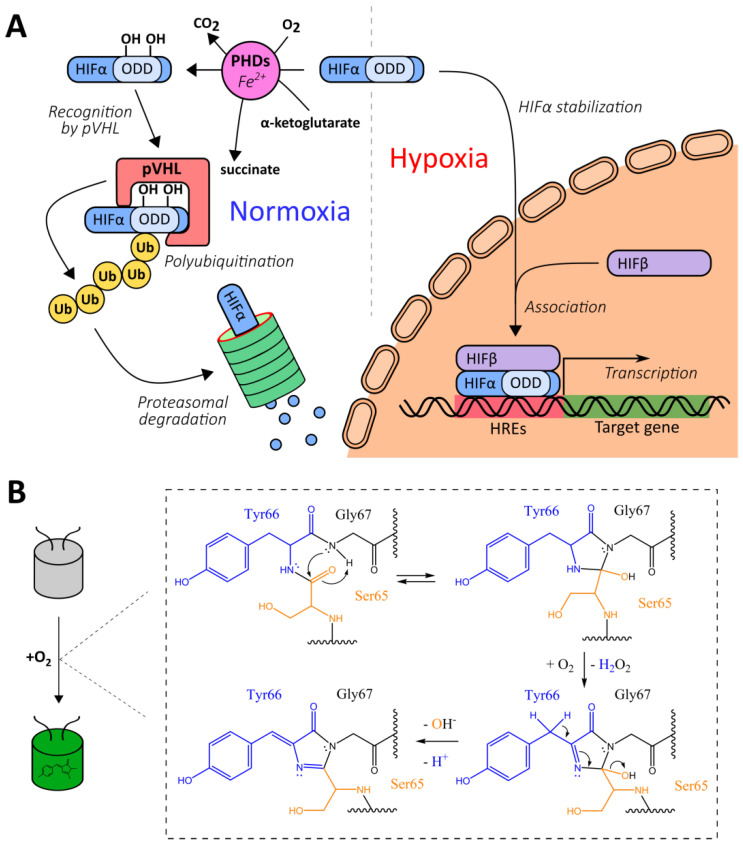
Some natural platforms for genetically encoded oxygen reporters’ development. (**A**) A simplified scheme of hypoxia-inducible factor (HIF) signaling system. (**B**) A simplified scheme of chromophore maturation in green fluorescent protein (GFP)-like fluorescent proteins (FPs).

The development of genetically encoded reporters requires the utilization of sensor domains from natural proteins containing molecular interfaces that have emerged in the course of evolution to specifically detect the biochemical parameter of interest. Most genetically encoded oxygen reporters are engineered on the basis of the hypoxia-inducible factor (HIF) system, which is a conserved signaling pathway in animal cells that is activated during hypoxia (Figure 1A) [35]. The HIF protein is a transcription factor consisting of α- and β-subunits. The latter is also known as “aryl hydrocarbon nuclear translocator”, and it is a constitutive nuclear protein that participates not only in oxygen sensing but in the detection of aromatic toxins [36]. In low oxygen conditions, the HIF heterodimer binds to hypoxia response elements (HREs) located in promoter regions of the target genes, modulating cellular energy metabolism, viability, vascularization, and other processes aimed to adapt to the lack of oxygen [35]. In mammals HIFα is encoded by three genes giving corresponding isoforms (HIF-1α, HIF-2α and several splice variants of HIF-3α) that differ by tissue distribution and biochemical properties (in particular HIF-3α4 variant lacks the known transactivation domains and apparently acts as HIF-1α negative regulator) [37]. HIFα subunits include the so-called oxygen-dependent degradation domains (ODDs), which contain critical proline residues (namely Pro402 and Pro564 for HIF-1α) [35]. When molecular oxygen is available, prolyl hydroxylases (PHDs) integrate oxygen atoms into these residues in the course of a reaction that requires 2-oxoglutarate as a third substrate and iron as an enzyme cofactor [38]. As in case of HIFα subunits, mammals have three PHD isoforms encoded in their genomes: PHD2 is the most wide-spread variant while PHDs 1 and 3 are more tissue-specific [38]. They also differ by their regulation patterns and selectivity for different HIFα isoforms. After PHD-depended hydroxylation, modified ODDs are recognized by a product of the von Hippel–Lindau tumor suppressor gene (pVHL), which is part of a multiprotein complex that includes elongin B, elongin C, Rbx1, and Cul2 [39,40]. This complex acts as an E3 ubiquitin ligase; in particular, it targets HIFα to proteasome-dependent degradation via a polyubiquitination step [39,40]. Thus, in normoxic conditions, HIFα is characterized by a very short half-life due to pronounced destabilization [35]. Two major groups of HIF system-based oxygen reporters are engineered on either HRE-driven reporter expression or on ODD-dependent reporter degradation. Another possible technique consists of coupling a reporter module to conformational changes of a chimeric protein that consists of an ODD and a hydroxyprolin-recognizing fragment of pVHL. 

Green fluorescent protein (GFP)-like fluorescent proteins (FPs), which are often used as reporters for genetically encoded probes, are actually oxygen-sensitive by nature. First, their autocatalytic chromophore maturation requires molecular oxygen as a substrate that acts as an oxidant for the generation of an elongated π-orbital system that is capable of absorbing visible light (Figure 1B) [41]. Second, in highly anaerobic conditions, the illumination of GFP by blue light results in a green-to-red photoconversion, known as anaerobic redding [42]. Both phenomena are implemented for measuring oxygen in living systems, giving rise to chromophore maturation-based probes and anaerobic redding imaging, respectively. 

Finally, in several studies, genetically encoded oxygen sensors based on haem-containing proteins were developed in which oxygen binding altered their ability to quench reporter module fluorescence or to act as a dark energy acceptor. In the next sections, we describe all of these tools from a mechanistic point of view and discuss their advantages and possible limitations. 

#### 2.1.2. Chromophore Maturation-Based Reporters

**Figure 2:**
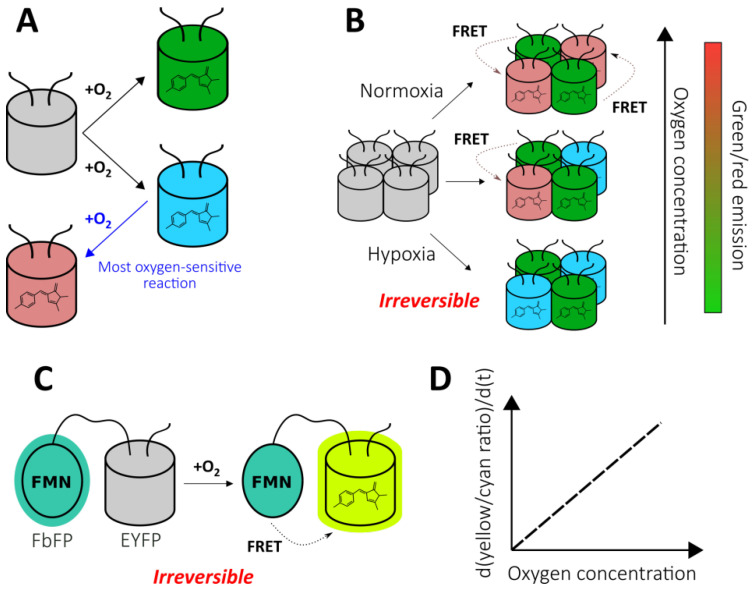
Chromophore maturation-based genetically encoded oxygen reporters. (**A**) Two competing pathways of DsRed chromophore formation. (**B**) The color dependence of nlsTimer probe on oxygen concentration during chromophore maturation. (**C**) The principal structure of fluorescent protein-based biosensor for oxygen (FluBO). (**D**) The time-dependence of FluBO yellow to cyan ratio growth on the available oxygen concentration.

As mentioned previously, FPs with autocatalytically formed chromophores require molecular oxygen for their maturation. Therefore, it is possible to utilize this feature for the development of oxygen reporters. The nlsTimer probe [43] was engineered based on DsRed FT, a non-aggregating tetrameric form of DsRed, that demonstrates two competing pathways of chromophore formation (Figure 2A) [44,45]. The first pathway generates green species, while the second pathway produces red-emitting molecules through a blue-colored form. In the course of DsRed FT expression, the blue fluorescence emerges first, followed by an increase in green and eventually red emission. The mature protein predominately emits in the red part of the spectrum due to extremely efficient Förster resonance energy transfer (FRET) between the green and red species [46]. Each maturation step requires an oxygen molecule that renders DsRed FT a natural oxygen sensor. In their work, Lidsky et al. demonstrated that the blue-to-red conversion efficiency strongly depends on the medium oxygenation (9–21% O_2_ concentrations were tested) [43]. Taking advantage of this observation, they developed the nlsTimer probe by fusing DsRed FT with a nuclear localization signal that enabled the confocal imaging of the oxygenation state of *Drosophila melanogaster* larvae cells during early development. The nlsTimer signal can be collected via two possible readouts: the ratio of green to red chromophores i) absorption or ii) fluorescence, which is generally convenient because the ratiometric character of the response increases sensor robustness toward differences in expression rates and cell shapes (Figure 2B). Parental DsRed is one of the most pH-tolerant FPs [47]; therefore, it is unlikely that the medium acidity would significantly affect response; however, this factor was not investigated in the original paper. On the one hand, in contrast to many other genetically encoded oxygen reporters, nlsTimer enables the observation of differences in oxygenation states when the oxygen concentration is above 5% (for example, it is known that pronounced accumulation of HIF-1α begins at oxygen concentrations of 5%, and it is expected that the sensors based on the HIF system inherit this feature); on the other hand, the performance of nlsTimer in more severe hypoxia has not been studied. The main drawbacks of nlsTimer include its slow maturation time (days) and irreversible character of the response. In the original study, the authors implemented a system consisting of *hs-GAL4* and *UASt-nlsTimer* constructs that allows the capture of oxygenation memory maps after heat shock in poikilothermic animal models, which reflect the average oxygen concentrations during chromophore formation rather than rapid changes [43]. The implementation of degrons could increase turnover of the probe, paving the way for repetitive imaging experiments (possible approaches are discussed in the context of HIF system-based reporters). 

As stated previously, nlsTimer has internal control, making ratiometric readout possible, that is absent in most FPs which demonstrate intensiometric decrease in fluorescence intensity due to disrupted maturation when O_2_ supply is insufficient. One strategy to overcome this obstacle is to fuse a GFP-like FP with an FMN-based fluorescent protein (FbFP). Such proteins are derived from bacterial or plant light-oxygen-voltage-sensing domains that have been engineered to make the non-covalently bound FMN fluorescent [48]. In this regard, FbFPs do not require molecular oxygen for maturation, and they are characterized by having low molecular masses, which could be useful in some situations. Fluorescent protein-based biosensor for oxygen (FluBO) was developed by fusing enhanced yellow fluorescent protein (EYFP) (λ_ex_ = 512 nm, λ_em_ = 530 nm) and FbFP (λ_ex_ = 450 nm, λ_em_ = 495 nm) with a short amino acid linker, placing the chromophores at a favorable distance for FRET (Figure 2C) [49]. The fluorescence intensity ratio (530 nm/495 nm), which is excited at 380 nm, depends on the degree of EYFP maturation because it enhances the efficiency of energy transfer by increasing the acceptor concentration. The EYFP variant used in this work has a pK_a_ of 5.2, and its emission is resistant to Cl^−^ concentration changes up to 100 mM; therefore, the medium acidity and Cl^−^ concentration are unlikely to affect FluBO readout [49]. The established fluorescence lifetime of mature FluBO in live *Escherichia coli* cells is 1.74 ns, compared to 2.73 ns of FbFP (according to biexponential and monoexponential analysis, respectively), indicating efficient FRET. If one imagines a portion of the FluBO protein that was synthetized under anoxic conditions, it might be expected that initially yellow fluorescence would be absent, and the fluorescence ratio would increase according to oxygen availability. Moreover, the molecules in which the EYFP chromophore had already been formed would create a strong background of efficient FRET. Therefore, although the FluBO ratio reflects the oxygen concentration at any given moment (which was proven by *E. coli* batch cultivation) the best way to read FluBO signal is to measure the rate of the ratio increase (dR/dt). The authors demonstrated that the rate of the ratio increase could even be calibrated to obtain absolute oxygen concentration values (Figure 2D) [49]. Productive FluBO performance in living systems—beyond imaging shifts from anoxic to normoxic conditions—apparently requires fast metabolism in order to produce new reporter molecules at a reasonable rate. It might be productive to complement the probe with a degron to increase biosensor turnover in order to reduce the background signal created by molecules that have already matured. Recently, the probe was adapted for fungal expression systems, namely *Saccharomyces cerevisiae* and *Candida albicans* [50]. 

#### 2.1.3. HRE-Based Reporters

**Figure 3:**
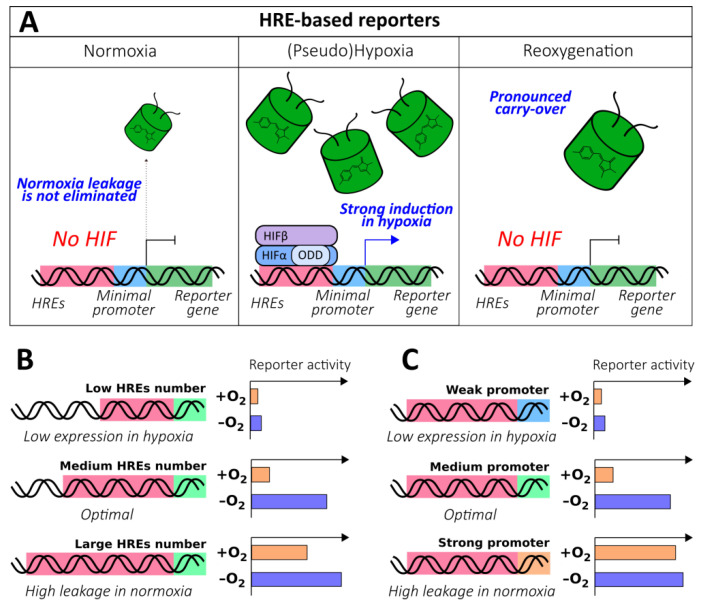
Hypoxia response element (HRE)-based genetically encoded oxygen reporters. (**A**) The general behavior of HRE-based oxygen reporters in normoxia, (pseudo)hypoxia and reoxygenation. (**B**) The dependence of HRE-based oxygen reporters’ behavior in normoxia and hypoxia on the number of HRE repeats. (**C**) The dependence of HRE-based oxygen reporters’ behavior in normoxia and hypoxia on the strength of the promoter.

The discovery of the HIF system, a highly conserved and specialized signaling pathway that evolved to detect molecular oxygen levels, made the construction of optical reporters on this platform possible. HREs were described prior to the elucidation of HIFα regulation on the molecular level; therefore, HRE-based reporters became the first tools of that kind. Many studies in this field were dedicated to the investigation of the parameters of the HIF system itself (several examples can be found in the following papers [51,52,53,54,55]); however, the lessons learned were also translated into the research and development of gene-directed therapy for heart diseases [56,57], cancer [58,59,60,61,62], and ischemic stroke [63,64] in addition to the visualization of hypoxia in tumor models [65,66,67,68,69,70,71,72,73], transgenic fruit flies [74], and fish [75]. All reporters from this group share a common structure that includes a promoter that drives the expression of the reporter unit under the control of HRE enhancers (Figure 3A). In hypoxic conditions, HIF signaling is activated, which leads to the increase of reporter activity. In this section, we describe the architectural details of these sensors that underlie their performance.

Because HREs endow these instruments with oxygen sensitivity, they represent the “heart” of the method. In general, an increase in the number of HREs leads to the elevated expression of the reporter in hypoxic conditions; this happens because when the oxygen concentration is limited, multiple HREs significantly enhance transcription by facilitating HIF binding. This feature seems to be attractive because it results in high levels of brightness; however, further addition of HREs usually results in low induction rates because in normoxic conditions, undesirable promoter leakage becomes more pronounced (Figure 3B). It is, therefore, important to adjust the system according to the investigation purposes. Thus, Boast et al. demonstrated that duplications of sequences from human enolase (ENO; 3HRE) and murine lactate dehydrogenase (LDH; 2HRE) genes resulted in 120- to 63-fold and 81- to 65-fold induction decreases, respectively, despite an overall increase in expression rates [76]. A similar trend was observed in a study by Shibata et al., where it was shown that 5 HREs from vascular endothelial growth factor (VEGF) gene elicited greater induction compared to 3 HREs (54- and 34-fold, respectively); however, the following addition of 5 more copies caused no further improvement (57-fold) due to saturation [59]. Fomicheva et al. demonstrated that in the double oxygen–sensing vector system (DOSVS), 6 HREs elicited a 3-fold induction increase compared to 2 HREs, despite an obvious increase in normoxic leakage [77]. A study by Takeuchi et al., which investigated the expression rates of luciferase reporters under the control of varying numbers of VEGF gene-derived HREs (from 2 to 12) depending on different hypoxic conditions (O_2_ concentrations from 1% to 16%) and different hypoxia durations (from 6 to 24 h), was in agreement with the general trends [78]. Some studies reveal more complicated patterns of HRE copy number influence, indicating that there might be other factors that affect HIF-mediated transcription, such as the three-dimensional alignment of HREs on DNA. Data published by Post et al. confirms that in the case of VEGF gene-derived HREs, induction saturation occurs at 5 copies, while the minimum statistically significant induction is detected at 3 copies; however, the authors observed a non-linear dependence of reporter expression rate on the erythropoietin (EPO) gene-derived HRE copy number [62]. 

Another important factor is the nature of the HRE repeats. HREs are found in different genes, and their exact structure directly affects both the leakage and induction rate of the reporter. Thus, in the same study by Post et al., almost all variants of reporters with EPO gene-derived HREs demonstrated low induction values in contrast to VEGF gene-derived HREs, mainly due to high activity in normoxia [62]. Boast et al. observed differences in both induction and expression rates between reporters with hENO and murine phosphoglycerate kinase (PGK) sequences (each containing 3 HRE copies) [76]. In hypoxic conditions, luciferase activity was greater than 5-fold higher for the mPGK reporter than for the hENO version. In their system, the highest induction rate was observed for the reporter that contained 4 hEPO sequences (8 HREs in total); however, this was achieved not due to high expression under hypoxia (moreover, it was approximately 3-fold lower compared to the mPGK reporter) but because of low leakage in normoxia [76]. In another study, it was shown that the HRE sequence from hENO1 was more powerful than other HREs known at that time (for example, those from human aldolase A (ALDA) and mLDHA) [51]. 

It is also important to remember that, despite the fact that the HIF system is highly conserved in animals, some regulatory differences still exist. In order to create an HRE-based reporter system for the fruit fly model, Lavista-Llanos et al. initially tested a sequence from mEPO gene that failed to induce hypoxia-mediated expression [74]. The absence of activity was apparently caused by the fact that HRE from mEPO gene is sufficient to bind HIF; however, it requires some additional coactivators that are not present in fly cells because the erythropoietin gene is not conserved in flies. When the authors implemented the enhancer from the mammalian LDHA gene that included two HREs and the cAMP-responsive element, which is well-conserved in flies, they detected an 8-fold induction of β-galactosidase in hypoxic embryos [74]. A similar issue is present in the context of HRE-based reporters’ specificity in different cells and tissues from the same species. Most studies have used tandem copies of HREs derived from enhancer regions of human EPO or VEGF genes that contain not only actual HIF binding sites but specific EPO or VEGF promoter sequences. It is not known to what extent they affect expression of these reporters in cells with different protein profiles. In order to overcome this obstacle, Zhou et al. suggested the implementation of minimal HIF binding sites to serve as regulatory elements in place of HRE sequences from real genes [54]. They were able to successfully develop reporters with hypoxia-sensing mechanisms that relied on the minimal HIF-1α and HIF-2α binding sites located in tandem (CGTGTACGTG). These dual HIF binding sites followed the same principles regarding copy number: a shift from 3 to 6 copies resulted in a significant increase in both the expression and induction (the latter changed from 5.8- to 33.4-fold); however, the addition of 6 more copies led to a 2-fold induction decline [54]. In another study, it was shown that the combination of the Egr1-binding site, a metal response element from the metallothionein gene, and 3xHRE from PGK1 gene resulted in significant hypoxia sensitivity improvement due to unknown synergistic interactions [79]. This combined enhancer was later implemented in the development of a hypoxia sensor [80]. It is worth mentioning that HREs act in a bidirectional way; they are capable of endowing oxygen sensitivity to 2 different genes located along their edges. Therefore, it is possible to control luciferase and β-galactosidase [62] or a double luciferase system [78] with a single enhancer element if needed. 

The expression and induction rates could be further adjusted by selecting an appropriate promoter (Figure 3C). In an early study by Shibata et al., the addition of the minimal E1b fragment containing a typical TATA box to *5xHRE-(385 bp VEGFP sequence)-luciferase* resulted in a 2-fold induction increase [58]. It was later shown that the substitution of (385 bp VEGFP sequence)-E1b for hCMVmp stimulated normoxic leakage; however, it increased the hypoxic induction rate 5-fold [59]. Boast el al. revealed that the combination of PGK-derived HREs with the SV40mp resulted in higher expression in hypoxic conditions at the cost of a more pronounced normoxic leakage compared to MLVmp [76]. In several studies, it was demonstrated that when very strong promoters were combined with HREs, they failed to respond to hypoxia. Thus, after preliminary tests, Cai et al. selected the SV40 minimal promoter, rather than CMV, to develop a therapeutic vector for delivering a matrix metalloproteinase-9/GFP fusion to the ischemic brain [63]. The EF-1α promoter, one of the strongest mammalian promoters, in combination with 5 VEGF-derived HREs was not as capable of inducing luciferase expression in hypoxia [59]. Certain issues could arise with tissue-specific delivery of the reporters. In a study by Phillips et al., the HRE-MLC-2v element provided selective luciferase expression in heart tissue; however, hypoxia-mediated induction was observed only when HIF-1α was overexpressed in parallel, suggesting that HIF had decreased access to such sequence [56]. In any case, performance of each HREs/promoter combination should be experimentally studied in the system of interest before implementation in physiological models.

It might be productive to use signal amplification systems to achieve better expression in some situations. Tang et al. developed a dual system consisting of a transactivator (*HRE-SV40-GAL4DBD-p65AD*) and a reporter (*6xGAL4UAS-E1bTATA-luciferase*) constructs [57]. This combination elicited up to a 60-fold increase in the expression rate in H9c2 cells under hypoxic conditions compared to a single plasmid reporter; however, the authors failed to obtain enhanced induction because of pronounced normoxia leakage. In the study by Lavista-Llanos et al., the authors implemented a combination of transactivator (*2x(2xHRE-CRE)-GAL4*) and reporter (*UAS-GFP*) constructs to investigate hypoxia during *Drosophila* development with better sensitivity, particularly in the presence of the cuticle [74]. It seems that to achieve both high expression and induction, it is favorable to use relatively weak promoters in the transactivator construct to avoid normoxia leakage, and this method could be further upgraded by the addition of ODD to the transactivator protein (discussed in the context of combined reporters). 

Another possible strategy of induction enhancement involves the destabilization of reporter mRNA in an oxygen-dependent manner. Many genes coding for cytokines, growth factors, and proto-oncogenes contain AU-rich elements in the 3’-untranslated regions of their mRNA [81]. The HuR protein has been shown to bind these sequences, stabilizing corresponding molecules in hypoxic conditions [81]. Boast et al. demonstrated that after the addition of the hypoxia stability region (HSR) from VEGF to the 3xHRE-luciferase reporter, the induction ratio in T47D cells increased 2-fold without impacting basal expression in normoxia [76]. A controversial observation is found in another study, where HSR from VEGF reduced luciferase activity both in normal and hypoxic conditions; however, destabilization was more pronounced in the former, conveying an overall induction improvement (from 131- to 191-fold) [59]. 

Luciferase and FPs represent general reporter units that can be coupled with HRE-based imaging systems. Their corresponding advantages and limitations are discussed later in the context of combined reporters. As HRE-based systems rely on reporter concentration increase during hypoxia, their signals are intensiometric by nature, which leads to possible artifacts attributed to cellular motility and expression heterogeneity. Moreover, hypoxia itself induces translational arrest and affects transcription profiles that could shift reporter signals to the undesired direction [82]. Thus, *Drosophila* embryos expressing β-galactosidase under HREs control demonstrated less intense sensor activity at 1% oxygen compared to normoxic controls [74]. It is, therefore, favorable to utilize a second reporter under the regulation of a stable promoter as a reference for signal normalization. GFP/DsRed [66,67,83,84] or firefly luciferase/*Renilla* luciferase [85] pairs can serve as examples of such an approach. However, in the latter study, it was demonstrated that the interpretation of such controls might become ambiguous. When Doran et al. expressed *Renilla* luciferase under a set of constitutive promoters (TK, SV40, CMV) in various cell types (Cos7, NIH3T3, Rcho-1), they observed significant increases in reporter activity induced by oxygen depletion [85]. This effect depended on the promoter-cell line combination; thus, in Rcho-1 cells, all 3 constructs demonstrated induction, and in Cos-7 and NIH3T3 cells, only SV40 and CMV responded, respectively. They next revealed that this phenomenon was HIF-1α-independent, as hypoxia mimetics (CoCl_2_ and desferrioxamine (DFO)) failed to reproduce the observation. Therefore, when they switched from the normalization protocol to the split transfection procedure, HRE-regulated firefly luciferase performance was improved during hypoxia [85]. Such unpredictable expression changes might not only significantly reduce the induction ratio of the reporter but create heterogeneity in results collected from different cells and tissues, independent of real oxygen concentrations. In some studies, the utilization of cap-independent internal ribosome entry sites in the development of HRE-based reporters has been suggested [76]. 

It is possible to modify reporter units in HRE-based systems to achieve specific goals. Bioluminescence resonance energy transfer (BRET) provides a range of advantages for in vivo imaging, namely, avoiding the concomitant excitation of the acceptor and the photobleaching of the donor. Moreover, this technology does not induce cell autofluorescence, and when the red-shifted acceptor is implemented, it becomes convenient for deep tissue imaging. Iglesias et al. engineered a chimeric reporter unit consisting of mCherry and firefly luciferase fused with a short amino acid linker [80]. The probe enabled the in vivo imaging of murine xenografts co-transfected with the HIF-1α-bearing activator vector [80]. HRE-regulated FPs were combined with PET enzymes, namely, xanthine phosphoribosyltransferase (XPRT) [83,84] or herpes simplex virus type 1 thymidine kinase (HSV1-TK) [70,83,84,86], which metabolically entrap radiolabeled xanthine or nucleotide analogs, respectively. Such systems allow for the visualization of hypoxia by both fluorescence microscopy and PET. In other studies, suicidal gene therapy enzymes such as cytosine deaminase, uracil phosphoribosyltransferase, and virus 1-thymidine kinase acted as fusion partners that potentially allows for the investigation of tumor treatment dynamics using fluorescence approaches [60,61]. 

Reporter systems for multiparameter imaging that include HRE-regulated FPs could be developed. The HypoxCR reporter consists of *2xHRE-GFP-PEST* and *pCMV-mCherry-geminin* sequences, and it allows researchers to visualize cellular oxygenation states and proliferation activity [68]. Hypoxia induces an increase in green fluorescence, while red emission is controlled by geminin that is stable only during S-G_2_M phases of the cell cycle. Using the probe in 2-photon imaging, Le et al. demonstrated the existence of different cellular populations in HEK293T xenografts [68]. Before moving to the next section, we would like to highlight the fact that almost all HRE-based reporters suffer from significant carry-over that results from a relatively high stability of reporter units. Thus, the GFP half-life is approximately 24 h, depending on the expression conditions [87,88]. HypoxCR engineering required much smaller value for the reporter unit to provide better resolution of cellular subpopulations. The authors tested different ODDs derived from HIF-1α (401–603 amino acids or 530–603 amino acids); however, they did not work well in their system. In contrast, the addition of the PEST degron from ornithine decarboxylase, a sequence enriched for proline, glutamate, serine, and threonine, significantly improved performance. In another study, a similar approach was implemented to improve temporal resolution of imaging with HSV1-TK-GFP fusion, leading to reporter half-life of approximately 3 h in contrast to the initial probe that was stable during the observation period [86]. 

#### 2.1.4. ODD-Based Degradation Reporters

**Figure 4:**
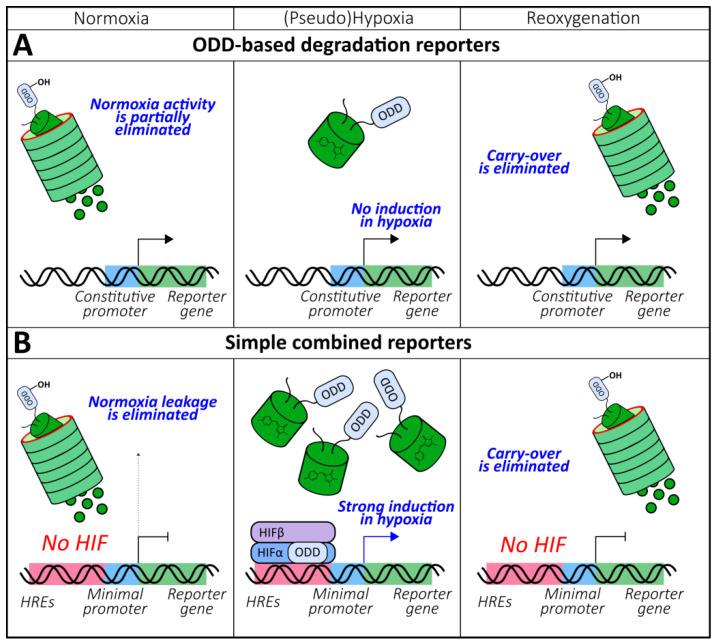
The general behavior of (**A**) oxygen-dependent degradation domain (ODD)-based degradation and (**B**) simple combined genetically encoded oxygen reporters in normoxia, (pseudo)hypoxia and reoxygenation.

As described previously, PHDs represent the oxygen-sensitive module of the HIF-signaling system. PHD-mediated hydroxylation of ODDs promotes the interaction of the pVHL protein with HIFα-subunits, driving the latter to the proteasomal degradation pathway. It therefore becomes possible to develop reporters that demonstrate oxygen-dependent behavior, which results from the incorporation of ODDs. All probes from this group represent destabilized proteins that are under the control of constitutive promoters, which are capable of decreasing signal intensity in the presence of molecular oxygen (Figure 4A).

The simplest approach in this field is to fuse an optical reporter with an intact HIFα protein, which can be found in several studies [55,69]. Despite being useful for HIF signaling investigation, chimeric proteins of this structure are not the best options for noninvasive physiological imaging due to the preserved transcriptional activity of HIFα. Apart from the fact that the increased concentration of HIF apparently affects the expression of the genes involved in cellular metabolism, HIF activates the negative feedback loop inside its signaling system via the activation of PHD2 and PHD3, among which the first is considered to be the major contributor [52,55]. Bagnall et al. established that in HeLa cells under reoxygenation, HIF-1α-EGFP degraded faster than ODD-GFP, which corroborates the described reasoning [55]. The data obtained by Lehmann et al. during C51 allograft imaging revealed that normalized photon counts coming from the HIF-1α-luciferase increased until the 9th day after which a pronounced activity decline was detected [69]. The authors hypothesized that such an observation could be explained in the terms of the negative feedback loop activation during hypoxia progression in the tumor cells [69]. Although the reporters without HIFα transactivation domains are not capable of enhancing this machinery, the described dynamics apparently are common to all ODD-based reporters due to natural HIF-1α accumulation, given that after reoxygenation, the ODD-GFP half-life in C6 glioma cells declined from 6 h [87] to 3 min [88]. Moreover, it seems that for obvious reasons, HRE-based systems suffer from the same shortcoming (as discussed later in the context of HIF system-based reporters’ limitations). 

The temporal behavior of ODD-based reporters depends on the exact structure of the destabilizing domain and, possibly, on the fusion partner. By implementation of the anti-Hyp564 antibody, D’Angelo et al. were capable of detecting hydroxylated ODD-GFP but not HIF-1α species in C6 glioma normoxic cells, which indirectly indicated that the former had longer half-lives [88]. It is not known to what extent the chemical structure of GFP contributes to this phenomenon; however, the absence of Pro402 in the utilized ODD represents an alternative explanation [88]. It is known that the C-terminal ODD, which is present in all ODD-based reporters, is sufficient for pVHL recognition and its subsequent degradation [89]; however, several diseases have been connected to altered PHDs selectivity supporting the importance of both Pro residues in adjusting HIFα dynamics in vivo [90]. The Pro402 and Pro564 residues of HIF-1α demonstrate different oxygen sensitivities, which is reflected in the more pronounced hydroxylation state of the latter under mild hypoxia [91]. Given that PHD3 is characterized by greater selectivity for this residue compared to PHD1 and PHD2 [92], one should expect that the tissue-related expression patterns of PHD isoforms would affect ODD-based reporters performance.

Certain amino acid substitutions in ODDs affect their affinity for PHDs that paves the way for the tuning of the dynamic properties of the chimeric reporters. ODD-luciferase reporter with an Ala substitution at Tyr565 showed a 3- to 4-fold higher steady-state level of activity in SH-SY5Y neuroblastoma cells in contrast to the wild-type construct, confirming the role of this residue in the ODD-PHD2 interaction [93]. In addition, the Pro567Ala substitution that is known to decrease ODD affinity for PHD3 demonstrated a smaller effect, which could be attributed to the fact that PHD3 is thought to be a minor isoform in SH-SY5Y cells [93]. Theoretically, it is possible to increase the oxygen sensitivity of ODDs by rational or random mutagenesis, creating degrons with reduced carry-over; however, we were unable to find any examples in the literature. ODDs can be derived from various HIFα isoforms, and their lineage plays an important role due to the differences in the temporal behavior of the parental proteins. Thus, a comparison of HIF-1α with HIF-2α reveals that the second is not only hydroxylated at slower rates by both PHD1 and PHD2 [94], but that both isoforms demonstrate different patterns of interactions with PHDs. Thus, PHD2 has more influence on HIF-1α than on HIF-2α, while PHD3 acts in the opposite manner [95]. To investigate the effects of ODD lineage on reporter functioning, Smirnova et al. developed a set of probes with degrons derived from HIF-1α, HIF-2α, and HIF-3α [96]. These reporters differed in normoxia leakage and induction rates in the presence of PHD inhibitors. In particular, ODD(HIF-2α)-luciferase showed the highest steady state but was better at distinguishing compounds with similar structures and elicited the best induction rate [96]. This was in agreement with the work by Bagnall et al., where increased HIF-2α-GFP fusion levels in normoxia were observed [55]. As ODD-based reporters rely on naturally present cellular enzymes for their functioning, phylogenetic relationships should be considered. Thus, Okamoto et al., in the course of directed gene therapy for canine breast tumors development successfully implemented reporters based on human ODD because its amino acid sequence is identical to canine ODD [97]. In another study, it was shown that the ability of PHD from *Drosophila* to recognize proline-containing peptides derived from human HIF-isoforms was reduced by 20–50% when compared to those from Sima (*Drosophila* homolog of HIF-1α) [98]. However, despite the observed difference, the authors used human ODD for the investigation of tracheal responses to hypoxia. Later, a reporter based on Sima-derived ODD was engineered [99,100].

Similar to HRE-based reporters, the strength of the promoter plays an important role in ODD-based reporters’ development. The general trend remains the same—weak promoters provide insufficient expression levels, while strong variants lead to extremely high normoxia leakage with significantly reduced induction (additional information might be found in the next section). Implementation of dual expression systems (for example, *(tissue-specific promoter)-GAL4* and *UAS-reporter*) is also possible [98,99]. In some research, the same reporter molecules without ODDs have served as controls for oxygen-independent perturbations in protein stability, transcription, and translation rates [69]; however, versions with critical proline residues substituted with alanine [93,96] may perform better due to potential ODD PHDs-independent influence. As ODD-based systems function on the basis of intensiometric signal, the expression of a stable reporter of a different color provides possibility of signal normalization in the course of a single experiment [99,100]. 

Based on general considerations, one should expect that when compared to HRE-based systems, ODD-based versions would detect hypoxia-to-normoxia shifts more accurately because of the destabilized reporter unit, which would result in less pronounced carry-over. On the other hand, the absence of a hypoxia-mediated mechanism of induction could slow their response during the normoxia-to-hypoxia shift. However, in some studies, similar patterns of performance have been observed. Lehmann et al. compared the behavior of reporters from both groups in the C51 allograft model, revealing a very similar temporal pattern of activation with very modest alterations [69]. 

ODD-based reporters have significantly contributed to our understanding of HIF signaling and facilitated the development of targeted gene therapy [97]. They have allowed for the creation of efficient PHDs inhibitors screening systems in cellular models that are free from the artifacts characteristic of in vitro assays attributed to the low activity of the purified PHDs, possible iron-overload during such protocols, and the insufficient affinity of substrate peptides compared to HIFα subunits [93]. Transgenic mice expressing ODD-luciferase [101] were used to investigate carbon tetrachloride-induced liver injury [102] and the hypoxic state of breast tumors during development and treatment [103]. GFP-ODD(Sima) allowed for the visualization of physiological hypoxia in the developing *Drosophila* brain and revealed region- and cell type-attributed patterns [99,100]. A protocol for imaging spontaneously occurring tumors hypoxia in immunocompetent ODD-luciferase-bearing transgenic mice is available [104]. 

#### 2.1.5. Combined Reporters (Chromophore Maturation, HRE-Mediated Expression, ODD-Mediated Degradation)

The insufficient temporal characteristics of HRE- and ODD-based reporters stimulated the development of instruments with more desirable kinetic properties. The simplest solution is to place an ODD-based reporter under the control of HREs (Figure 4B). Thus, Harada et al. upgraded the 5xHREs-luciferase system by fusing the reporter protein with HIF-1α ODD [105]. The described approach provided two main advantages over the original version. First, the half-life of the luciferase bioluminescence in HeLa xenografts decreased from 9 h to 17 min due to the eliminated carry-over during reoxygenation, which allowed for the real-time imaging of this parameter [105]. Secondly, the dual control, which prevented the activity of the reporter in normoxia, improved the induction rate by 2 orders of magnitude due to minimized leakage [105]. This approach allowed for the detailed investigation of the interplay between Akt/mTOR and HIF signaling during tumor reoxygenation after radiotherapy [106]. Later, transgenic mice bearing this reporter were created and mated with rasH2 Tg mice, who develop spontaneous tumors after N-methyl-N-nitrosourea treatment, to investigate hypoxia in vivo during oncogenesis [107]. A protocol was published that described the visualization of the hypoxia dynamics of breast tumor metastases in a mouse brain in vivo using HRE-ODD-luciferase [108]. In other studies, mCherry was implemented as a reporter unit that is favorable for deep tissues imaging [109]. Fluorescence microscopy of MDA-MB-231 xenografts revealed the oxygenation states of tumors with single-cell resolution [109,110]. In both articles, a stable GFP construct under the control of a constitutive promoter was used for signal normalization. A test system where HRE-regulated ODD-GFP was expressed in near-haploid KBM7 human cells was developed to search for the genes involved in HIF signaling [111]. Implementation of this approach revealed the role of the 2-oxoglutarate dehydrogenase complex [111] and V-ATPases [112] in PHDs regulation. 

**Figure 5:**
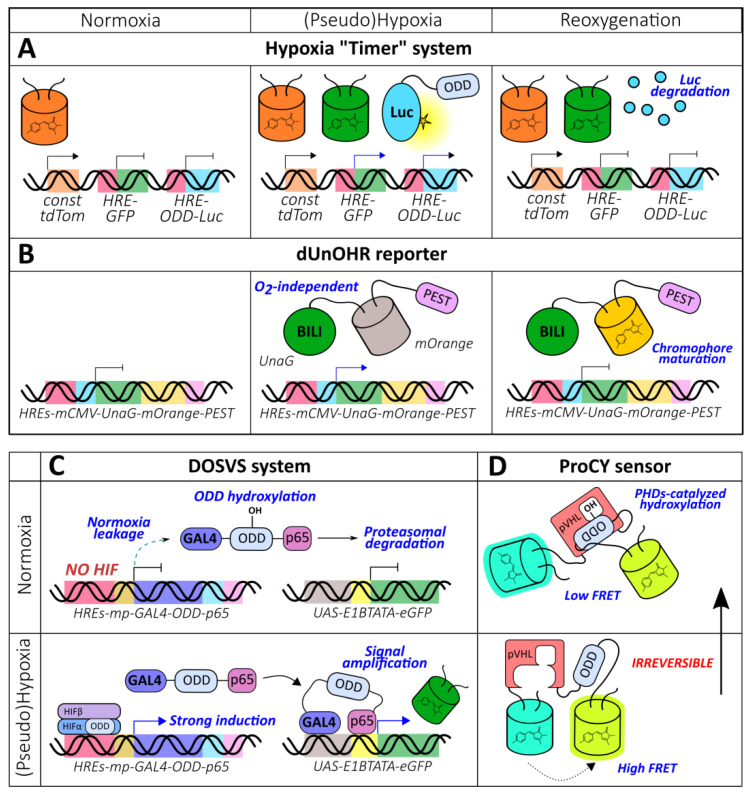
More complex genetically encoded oxygen reporters. (**A**) Hypoxia “Timer” system and (**B**) dUnOHR reporter that allow for distinguishing normoxic, hypoxic and reoxygenated cells on the basis of the observed optical reporters combination. (**C**) Dual oxygen-sensing hypoxia responsive sensor/effector viral expression system (DOSVS) that provides high induction rate due to eliminated normoxia leakage and signal amplification during hypoxia. (**D**) ProCY is an ODD hydroxylation-based reporter constructed according to a classic genetically encoded Förster resonance energy transfer (FRET)-biosensor design.

Reporters that are more complex than just a combination of HREs and ODD have been described in the literature. Danhier et al. developed a “timer” system capable of distinguishing normoxic, hypoxic, and reoxygenated cells (Figure 5A) [113]. Their approach outlined the implementation of 3 constructs: HRE-GFP, HRE-ODD-luciferase, and tdTomato under the control of a constitutive promoter. The measured half-lives of GFP and ODD-luciferase in PC3 cells expressing this reporter were 15 h and 34 min, respectively [113]. Thus, in normoxic conditions, only red fluorescence is visible while hypoxia triggers green fluorescence and the bioluminescent signal of luciferase. Luciferase is degraded during reoxygenation, which results in the loss of its activity. The authors succeeded at visualizing cellular populations that differed in their oxygenation states in subcutaneous and orthotopic prostate tumor xenografts with the “timer” system [113]. The described system possesses several drawbacks. The tdTomato signal is not entirely constitutive; it was shown that in hypoxic conditions, its maturation was likely affected, which led to a red fluorescence increase during reoxygenation [113]. This effect was not reproduced under hypoxia mimetic (CoCl_2_) treatment, confirming this interpretation [113]. Prolonged hypoxia (1% O_2_, more than 8 h) also resulted in a decline in red fluorescence [113]. The described behavior was not observed for GFP, which reflects that oxygen requirements for maturation can vary [113]. Another technical limitation could arise when comparing GFP and ODD-luciferase signals because fluorescence is more affected by tissue attenuation during excitation and emission than bioluminescence.

To overcome the potential artifacts caused by the disrupted chromophore maturation of GFP-like FPs in hypoxic conditions, Erapaneedi et al. developed a probe [114] based on UnaG, which is a small acidic protein belonging to the fatty acid binding protein family (Figure 5B) [115]. UnaG non-covalently binds bilirubin with subnanomolar affinity and activates its fluorescence [115]. Its optical properties, therefore, do not depend on the oxygen supply. The authors engineered several destabilized constructs by fusing UnaG with different degrons (ODD, PEST, or both) and placed all constructs under the control of the 5xHRE-CMVmp sequence. All probes increased their fluorescence under hypoxia; the half-lives after reoxygenation were 7.8 h, 5.5 h, and 30 min for PEST-, ODD-, and dual-based variants, respectively [114]. Despite eliciting the lowest carry-over, the dual reporter demonstrated the minimal induction at the cost of a high degradation rate, even in hypoxic conditions. When dEGFP-PEST was expressed in the same system, it failed to demonstrate induction in hypoxia; and after reoxygenation, a rapid increase in fluorescence followed by signal extinguishing was observed [114]. This observation can apparently be attributed to the accumulation of immature molecules that generated chromophores when molecular oxygen became available. Next, the authors combined HIF system- and chromophore maturation-based approaches by fusing UnaG-PEST to mOrange with a short amino acid linker. The resulting reporter, named dUnOHR, displays “timer” behavior: transfected normoxic, hypoxic, and reoxygenated cells are colorless, green, and green-orange, respectively [114]. One possible limitation of UnaG-based probes is that the utilization of the external chromophore could hamper imaging when the bilirubin supply is insufficient. 

Fomicheva et al. demonstrated that the implementation of ODDs can upgrade dual reporter systems [77]. The DOSVS consists of a transactivator and a reporter construct (Figure 5C). The second is represented by the *6xUAS-E1bTATA-(EGRP or luciferase)* sequence, which is transcribed in the presence of the GAL4-p65 chimeric protein. The authors hypothesized that the addition of ODD to this protein would efficiently destabilize it in normoxia leading to low leakage. When GAL4-ODD-p65 was controlled by a weak Myh6 promoter, the expression level in normoxia was indeed low; however, both induction and expression during hypoxia were very modest. The application of a strong CMV promoter resulted in high reporter activity, but oxygen-dependent behavior was lost [77]. These data indicate that ODDs alone are not capable of endowing oxygen sensitivity in the context of high expression rates. The final transactivator sequence included GAL4-ODD-p65 under the control of 6xHREs-SV40mp [77]. The combination of HREs and a relatively weak promoter provides low transcription efficiency during normoxia, while a small amount of leakage is eliminated by ODD-mediated destabilization. However, when oxygen becomes limited, the accumulating activator induces pronounced transcription of the reporter gene. Fomicheva et al. delivered DOSVS to the heart of adult rats via ultrasound-guided intramyocardial injection [77]. Following either chronic left anterior descending artery ligation or incubation of the animals in a hypoxic chamber confirmed the probe’s functionality (maximum induction rate of approximately 700-fold). 

We will now discuss the biochemical nature of optical reporters that could be coupled with HIF system-based probes. The implementation of luciferases and the implementation of FPs represent two primary strategies in this field. In general, bioluminescence does not require excitation light; therefore, visualizing deep tissues becomes more convenient; moreover, using bioluminescence provides better contrast and signal-to-noise ratio due to the absence of cell autofluorescence. However, compared to FPs, imaging with luciferase results in a loss of the resolution of the final data. Luciferin administration is an additional procedure that could lead to a number of imaging artifacts that one should be aware of. Luciferin metabolism differs between individuals and also differs within the same organism depending on its physiological condition (for example, respiratory rate or body temperature) [104]. Moreover, it has been shown that some anesthetics act as luciferase inhibitors and/or affect bioluminescence imaging due to their hemodynamic effects [116]. Experiments with transgenic ODD-luciferase mice have revealed high background signals in regions such as the kidneys, abdominal fat pad, and thyroid gland [101,103]. Despite that these observations for some reason were not confirmed by imaging of HRE-ODD-luciferase mice [107], it has been suggested that the kidneys and thyroid are physiologically hypoxic regions. However, the increased signal in the fat pad seems to result from peripheral vasoconstriction and a transient ischemia during anesthesia [104]. This phenomenon can be reduced by maintaining the mouse under anesthesia in a warm environment in dorsal recumbency for several minutes [104]. In the context of hypoxia imaging, it is worth mentioning that ischemic tissues often suffer from insufficient blood supply, which might affect luciferin accessibility. For instance, Goldman et al. observed low activity of ODD-luciferase reporter in the central nodules of solid tumors [103]. They suggested that, in part, this was caused by both the development of a necrotic core that lacked viable cells and limited luciferin access to this region [103]. 

As discussed previously, GFP-like FPs require molecular oxygen for their maturation, which might decrease the signal intensity in prolonged hypoxia and/or produce fluorescence outbursts during reoxygenation that mimic hypoxia-specific induction. Appropriate controls must be implemented to disentangle these processes. Luciferase reaction also requires molecular oxygen. In a study by Moriyama et al., 9L cells expressing firefly luciferase demonstrated strong enzyme activity dependence on oxygenation conditions [117]. The shift from 21% O_2_ to 0.2% O_2_ led to almost a 2-fold decrease in photon counts. Additional experiments suggested that the ATP level during hypoxia was the limiting factor in this model [117]. However, in another study, HeLa cells transfected with *Renilla* luciferase that did not use ATP as a substrate showed reduced reporter activity in hypoxic conditions [118]. Moreover, given that the firefly luciferase Km for oxygen is approximately 10 µM [119], and the corresponding value for PHDs is approximately 200 µM [92], it is unknown whether overexpression of luciferase might affect oxygen metabolism (namely, its redistribution between different enzymes). If FPs are selected as reporter units, red versions are favorable because they are excited at longer wavelengths, which have greater tissue-penetrating abilities and lead to decreased phototoxicity and background autofluorescence. To date, there is a number of studies that have implemented red FPs as reporter units for HIF system-based probes [60,72,109,110,114,120]. However, it is important to keep in mind that they require more oxygen molecules for chromophore synthesis, and experimental evidence exists suggesting that when oxygen is limited, their maturation in vivo proceeds longer than for their green counterparts [121,122]. On the other hand, in a study by Misra et al., it was shown that *Drosophila* embryos reared under 60% O_2_ demonstrated decreased emission of mRFP, which was under the control of a constitutive promoter [99]; therefore, the oxygen supply in vivo is not a straightforward acting factor. 

Finally, when HIF system-based reporters are preliminary tested in cellular cultures before physiological studies, hypoxia mimetics are often implemented to proof the workability of the approach. The induction promoted by these compounds is much greater than the value that could be observed even in highly hypoxic conditions [65,84]. The dynamic range of the reporter, therefore, might be dramatically overestimated. This method is not also capable of revealing artifacts attributed to the disruption of chromophore maturation [113]. Moreover, as was demonstrated by Smirnova et al., different hypoxia mimetics show unequal patterns of HREs activation depending on the exact composition of the latter [93]. 

#### 2.1.6. ODD-Based Hydroxylation Reporters

A large group of genetically encoded fluorescent indicators (GEFIs) is represented by FRET probes, in which two fluorescent proteins are fused to the edges of a sensory unit capable of detecting the molecular event of interest. When the value of the measured parameter changes, the sensory unit undergoes conformational rearrangement that affects the distance and/or the angle between the two chromophores of the fluorescent proteins, leading to shifts in the energy transfer efficiency. The described technology has recently been incorporated into oxygen reporters’ development. The ProCY biosensor consists of enhanced cyan FP (ECFP) and yellow FP YPet fused by a linker that includes fragments of pVHL (60–154) and HIF-1α (556–577) separated by 21 amino acid GS-rich sequence (Figure 5D) [123]. In the presence of molecular oxygen, Pro564, which is located inside the HIF-1α fragment, becomes hydroxylated by cellular PHDs, transforming HIF-derived sequence into a binding partner for the pVHL-derived domain. The FRET ratio of the probe decreases with a maximum amplitude of 168% during in vitro incubation with a catalytically active fragment of PHD2 [123]. The dose-dependent behavior attributed to the oxygen concentration has also been observed in living transfected HEK293T cells. The response was not altered when PHD2 was overexpressed, indicating that the activity of the natural enzymes was not the limiting factor in the studied system [123]. The observed changes of the signal completed in 30-40 min in vitro and 2–3 h in vivo after reoxygenation likely due to the slow oxygen dissolution and diffusion in the culture medium; however, the authors hypothesized that the existence of the oxygen transport systems in living organisms would improve kinetics [123]. An inactivated version of the probe with Pro564 and Pro567 residues substituted with Ala (ProCY-N) could be implemented as a control in physiological models [123]. One of the main limitations of ProCY in the context of real-time imaging is that prolyl hydroxylation is considered to be an irreversible modification; thus, the sensor reaches a steady state only after 48 h post hypoxia onset [123]. Fusing ProCY with degrons could improve its temporal resolution; however, strong expression is needed to achieve the production of novel reporter molecules. 

#### 2.1.7. General Limitations of HIF System-Based Reporters for Hypoxia Visualization

Now, we briefly discuss the main potential limitations of HIF system-based reporters in oxygen visualization in vivo. In general, many studies have reported that the activity of these sensors (and/or HIF-1α-staining) does not correlate well with other hypoxia probes such as pimonidazole [60,66,69,106] or fluoromisonidazole [69]. In particular, this has often been observed during tumor reoxygenation after radiation treatment and is not attributed to the decreased dye accessibility proven by perfusion markers [60,106]. On the one hand, these types of artifacts could be caused by the very slow kinetic properties of the HIF system-based reporters, which often need hours or, at least, dozens of minutes to respond. In MDA-MB-231 xenografts, reporter-labeled tumor cells were detected near well-perfused blood vessels, which was apparently caused by their migration to these regions [109]. Possible tumor cell migration was also detected with the use of dUnOHR [114]. However, it seems that in many cases, pronounced carry-over cannot explain the discrepancy. Idovina et al. detected HRE-mediated reporter activity in small spheroids (<100 µm), and given that this value is the estimated diffusion limit of molecular oxygen [65], it is more likely that in this case, the observed phenomenon resulted from three-dimensional organization, mediated by some paracrine factors affecting cellular metabolism, signaling, and gene expression. 

In certain cases, the reporter activity could be mediated by processes that do not involve the stabilization of ODDs and, therefore, HIFα. Moeller et al. demonstrated that in hypoxia, 4T1 cells accumulated stress granules containing HIF-regulated transcripts; during reoxygenation, these mRNA were activated, which stimulated EGFP emission [66]. In another study, it was shown that PTEN-deficient cells had elevated HRE-luciferase activity that was unrelated to HIFα stabilization. Detailed investigation revealed that in this system, FOXO3a was exported from the nucleus, which prevented it from acting as a negative regulator of HIF coactivator p300 [53]. One would expect that similar artifacts might arise in other models. For example, it was shown that interleukin-8 was capable of inducing VEGF expression in HIF-1-deficient tumor cells [124]. 

The most pronounced oxygen-independent influence on HIF system-based reporters obviously arises from the fact that this pathway integrates biochemical information of a different nature. Harada et al. revealed that the upregulation of HIF-controlled genes (5HRE-ODD-luciferase) during tumor reoxygenation was partially attributed to the glucose-dependent activation of Akt/mTOR signaling, which stimulated HIF-1α mRNA translation with subsequent accumulation of the latter [106]. This is just a single example that was investigated using the discussed reporters. Various factors other than O_2_ are known to adjust HIF-1α concentration: insulin [125] and IGF-1 [126] are capable of increasing HIF-1α levels via posttranscriptional mechanisms; eIF2α phosphorylation inhibits HIF-1α mRNA translation [127]; RNA-Binding Proteins HuR and PTB act in the opposite manner [128]; and Hsp90 protects HIF-1α from pVHL-independent degradation [129]. 

In addition to HIFα, other system components integrate biochemical information. As discussed previously, a negative feedback loop exists in HIF signaling due to the HIF-mediated accumulation of certain PHD isoforms [55,88,130]. It has been suggested that this mechanism contributes to the fact that HIF system-based reporters demonstrate a decline in their activity during prolonged hypoxia [65,69]. It is also important to consider that the discussed PHDs upregulation is tissue-dependent [130]. Disruption of cellular iron and energy metabolism could affect PHDs activity. Thus, research using HRE-ODD-combined reporters revealed that the inhibition of V-ATPase led to decreased transferrin uptake and conversion of Fe^3+^ to Fe^2+^, which reduced PHD activity due to the lack of the reaction cofactor [112]. In another study, it was shown that the disruption of lipoate synthesis inhibited the 2-oxoglutarate dehydrogenase complex, triggering 2-oxoglutarate accumulation. The latter was subsequently converted into L-2-hydroxyglutarate, which acted as a PHD inhibitor [111]. It is well known that mutations in succinate dehydrogenase are linked to elevated levels of its substrate, which is also a potent inhibitor of prolyl hydroxylation [131]. Although these factors certainly play a significant role in genetic diseases and oncogenesis, it remains unclear if analogous events occur under normal physiological conditions. Moreover, it is useful to keep in mind that PHD isoforms demonstrate different tissue distribution and selectivity. Additional information about these enzymes can be found in the following reviews [38,132]. 

Potential artifacts can arise on the pVHL level. After two months post 7,12-Dimethylbenz(*a*)anthracene (DMBA) treatment, fluorescent cell clones were identified primarily in the retina, skin, gills, and fins of transgenic *vhl^+/−^ phd3::EGFP* fish [75]. The observed reporter activation in random cell populations suggested the DMBA-induced loss of the second functional gene copy rather than hypoxic conditions in these regions [75]. Genome sequencing analysis of the fluorescent tumors that developed in these organisms failed to find novel mutations in *vhl^+^* allele; however, real-time PCR revealed that in 4 of 5 tumors, the *vhl* mRNA concentration was significantly reduced compared to the control sample [75]. These experiments provide strong evidence that artifacts that are caused by pVHL depletion-induced pseudohypoxia can occur during in vivo oxygen imaging with HIF system-based reporters, especially in cancer models. 

The production of reactive oxygen species (ROS) in a system can also interfere with the described methods. Moeller et al. demonstrated that tumor antioxidant treatment after radiotherapy eliminated HIF-1 upregulation and, consequently, the reporter activity [66]. In another study, it was shown that reoxygenation of HeLa cells led to ODD-galactosidase protein stabilization, which diminished under catalase treatment, indicating the role of ROS [106]. In general, ROS are known to be important HIF signaling modulators that act via different pathways, and PHDs inhibition is suggested to be one of the primary mechanisms [133,134,135]. 

The quantitative interpretation of data obtained with the described reporters is additionally hampered because of tissue- and cell-specific differences in HIF signaling sensitivity. If zones of high signals are found in the model, auxiliary experiments are needed to reveal their connection either to a real difference in the oxygenation state or to a reduced threshold for HIF activation. As mentioned previously, ODD-luciferase transgenic mice show increased bioluminescence in their kidneys and thyroid glands; this phenomenon is explained by the theory that these organs apparently experience hypoxia under physiological conditions [101,103]. Transgenic *phd3::EGFP* fish embryos that were raised in low oxygen concentrations were characterized by more pronounced fluorescence in the CNS and pronephros, and the exact rationale for this is unknown [75]. In another study, transgenic *Drosophila* bearing a double reporter system (HRE-Gal4/UAS-GFP) showed constitutive signals in their salivary glands [74]. Moreover, when developing embryos were placed at 5% O_2_ concentration for 4 h, tracheal cells produced the greatest response despite their direct contact with air. In more severe hypoxic conditions (4% for 16 h), reporter activity was clearly observed outside the tracheae in tissues and organs such as the ectoderm, esophagus, gut, fat body, muscles, and gonads [74]. This observation was consistent with experimental evidence that tracheal cells are enriched with the dVHL protein [136]. By contrast, the ODD-GFP reporter did not respond to an oxygen concentration shift from 21% to 5% when expressed in the ectodermal cells of *Drosophila* embryos and larvae [98]. Overexpression of dVHL led to a 3.5-fold induction under the same experimental conditions, confirming that ectodermal cells failed to efficiently sense hypoxia because of the inefficient degradation of hydroxylated Sima [98]. Distinct cancer cell lines (786+VHL, A549, ME180, U251, and HeLa) are characterized by different emission intensities of EYFP (controlled by minimal HIF-binding sites) at the same oxygen concentrations that correlate with total HIF-1α and HIF-2α levels [54]. In particular, the higher reporter activity in ME180 cells over HeLa cells was shown to be dependent on increased hsp90 content, supported by the fact that treatment with 17-allylamino-demethoxygeldamycin, which inhibits ATP binding to hsp90, resulted in a decline in the induction rate [54]. Baccino-Calaci et al. established a quantitative relationship between GFP-SimaODD/mRFP-nls ratio in single cells of the developing *Drosophila* brain and the distance to the closest tracheole [100]. However, they found that some cells significantly deviated from the predicted pattern; neuroepithelial cells of the inner proliferation center and central brain ganglion mother cells demonstrated over- and under-valuated signals, respectively [100]. The authors also revealed that, in contrast to neuroblasts, optic lobe neuroepithelial cells had a more limited ability to respond under hypoxic conditions [100]. After reanalyzing cell type-specific Polymerase II DamID data [137], they hypothesized that it could be attributed to the fact that the transcriptome of neuroblasts is highly enriched in hypoxia sensing pathway genes [100]. 

The insufficient kinetic properties of HIF system-based reporters result not only in the poor temporal but in the poor spatial resolution of signals; therefore, they do not allow for the visualization of subcellular oxygen gradients. Moreover, it seems that even in the context of oxygen sensing, they better reflect the average oxygen availability to PHDs rather than the actual oxygen concentration. Thus, Hagen et al. demonstrated that in HEK293 cells incubated at 1% O_2_, the inhibition of mitochondrial respiration at any site of the electron transport chain led to pronounced HIF-1α destabilization [118]. These results were replicated in Hep3B, 143B, and HeLa cells; the phenomenon was not attributed to ROS emerging in the course of the treatment, as antioxidants failed to rescue this behavior [118]. Because NO donor DETA-NO and myxothiazol lowered the ODD-GFP concentration in hypoxia, the authors suggested that the inhibition of respiration redistributed oxygen between cytochrome oxidase and PHDs [118]. In this model, the activity of *Renilla* luciferase (O_2_-dependent) significantly increased under NO treatment at 1% O_2_, confirming that in hypoxia, when oxygen concentration is limited, competition between different enzymes indeed may affect the HIF pathway [118]. O’Hagan et al. reported that PGC-1-stimulated mitochondrial biogenesis in HeLa cells acted in the opposite manner; namely, it stabilized both HIF-1 and ODD-GFP due to elevated oxygen consumption in the presence of higher mitochondrial biomass [138]. Finally, ODD-containing probes obviously require physical contact with functional PHDs. When HEK293T cells, expressing either nls- or nes-ProCY, were cultured in normoxia, reduced FRET ratios (indicative of PHDs activity) were observed in the cytosol but not in the nucleus, suggesting that in this system, PHD activity was not equally distributed [123]. 

In conclusion, HIF system-based reporters have undoubtedly contributed to our understanding of both hypoxia and HIF signaling. However, because this system integrates a variety of biochemical stimuli other than oxygen concentration, they are much better probes for HIF signaling rather than for oxygen due to their inability to distinguish hypoxia from pseudohypoxia in certain cases. Because PHDs act as oxygen sensors inside HIF pathway, the distancing of the molecular switch that alters reporter activity from the hydroxylation reaction increases the number of possible information flows, while they are the ones which generate artifacts in the context of oxygen in vivo visualization. Therefore, we can trace the increase in ability to measure exactly oxygen in the HRE-reporters, ODD degradation-reporters and, finally, ProCY row. 

#### 2.1.8. Anaerobic GFP Redding-Based Imaging

**Figure 6:**
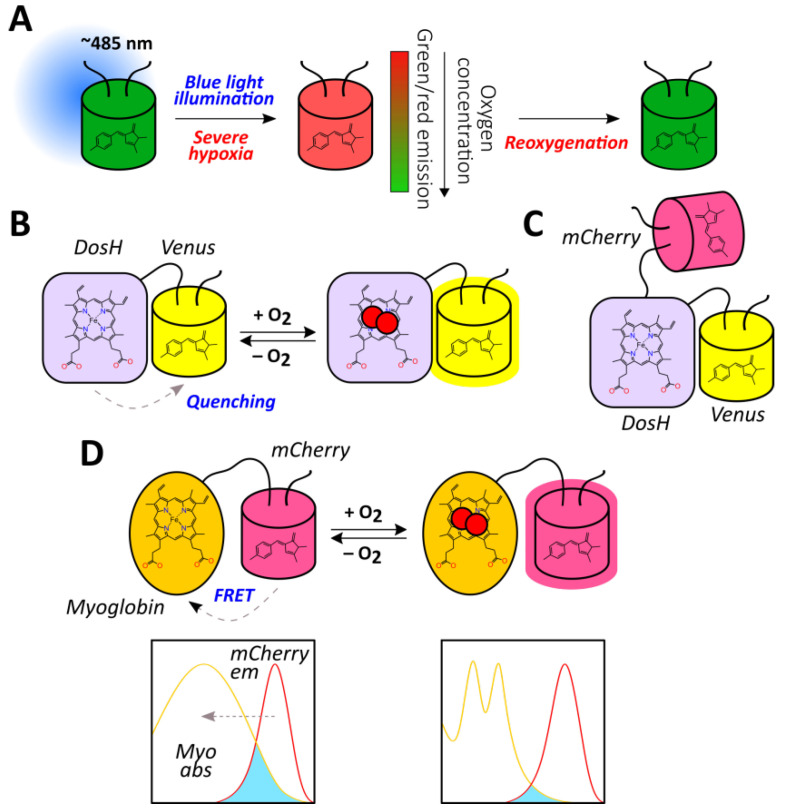
The methods for direct oxygen detection with the use of fluorescent proteins. (**A**) GFP anaerobic redding-based imaging. (**B**) The principal structure of ANA-Y probe where oxygen binding to the haem in DosH domain disrupts its ability to quench Venus fluorescence. (**C**) Ratiometric version of ANA-Y, named ANA-Q, which includes mCherry as the internal control for signal normalization. (**D**) The principal structure of Myo-mCherry probe where oxygen binding to the myoglobin haem lowers its ability to act as a dark acceptor for the excited mCherry chromophore during FRET.

Elowitz et al. discovered that in low oxygenation conditions, GFP illumination with blue light (475–495 nm) resulted in green-to-red photoconversion (Figure 6A) [42]. This phenomenon was observed for different GFP mutants both in purified proteins and in living bacterial cells when proteins or cells were incubated in the presence of oxygen scavenging systems or sealed in microscope slides for approximately 25 min [42]. To test whether GFP could act as an oxygen reporter in mammalian cells, Takahashi et al. induced green-to-red photoconversion at different oxygen concentrations in transfected COS-7 cells and cardiomyocytes from transgenic mice [139]. Blue light illumination in almost anoxic conditions led to the emergence of red fluorescence with a corresponding decline in the green channel which was consistent with the first report [139]. Such an approach allows for ratiometric imaging; thus, red/green fluorescence ratio of Hep3B cells expressing AcGFP1 in the cytoplasm after a pulse of blue light (220 s) was approximately 0.074 at 10% O_2_, 0.078 at 3% O_2_, 0.101 at 1% O_2_, and 0.220 at 0.001% O_2_ [140]. It is therefore seen that the most pronounced shift occurs when the oxygen concentration falls below 1%. Using purified protein, the authors demonstrated in vitro that the pH value of the medium (pH 6 to 8) did not affect the red/green ratio [140]. They visualized oxygen gradients in monolayer Hep3B cells covered by glass that became less rapid in the presence of KCN, suggesting that cellular respiration was an important contributor to their emergence [140]. When AcGFP1 was overexpressed in the mitochondria of cultured Hep3B cells, oxygen gradients were visualized in individual cells [140]. GFP anaerobic redding-based imaging is also applicable to more complex models; in one study, hypoxia imaging was performed in the ischemic livers and hearts of GFP knock-in mice; imaging took place during portal vein/hepatic artery ligation in the livers and ligation of the left anterior descending branch of the coronary artery in the hearts [141]. The described photoconversion is reversible because red fluorescence quickly disappears when oxygen becomes available [141]. Although it seems that this phenomenon allows for the direct visualization of oxygen, and the response is ratiometric by nature, such an approach has several drawbacks. First, anaerobic redding becomes pronounced only in severe hypoxia, which limits it implementation in many situations. Second, the exact mechanistic processes that underlie this behavior are still unknown, which hampers the prediction of potential artifacts. However, their discovery in the future may provide a basis for rational enhancement of the method including oxygen sensitivity adjustment.

#### 2.1.9. Haem Proteins-Based Reporters

To complete this subsection, we will discuss oxygen GEFIs based on haem proteins. Bacterial phosphodiesterase DosP is an enzyme with a catalytic domain that converts cyclic di-GMP to linear di-GMP [142]. Its activity depends on molecular oxygen concentration due to the presence of a haem-binding regulatory domain (DosH), whose structure has been solved [143]. Oxygen binding induces conformational rearrangement of the protein; however, it is relatively modest that hampers its utilization in FRET based biosensor development. On the other hand, DosH demonstrates pronounced Soret and Qy peaks at 425 and 560 nm that are shifted to 414 and 580 nm upon oxygen binding [144]. Therefore, it is possible to utilize the observed electron redistribution in the haem as a molecular switch that transfers information about its oxygenation state to a reporter module. Nomata et al. engineered a set of genetically encoded sensors by fusing FPs (Venus or EGFP) with DosH via an antiparallel coiled-coil linker (Figure 6B) [145]. The fluorescence intensity of ANA-Y (527 nm) and ANA-G (510 nm) probes increased upon oxygenation with maximum response values of 1.7- and 2.1-fold, respectively [145]. This behavior likely emerged from the Fe^2+^-induced fluorescence quenching of the reporter protein in the oxygen-free state. The measured K_d_ of ANA-Y for oxygen was 18 µM, which was comparable to the values of intact DosH (13–20 µM) published previously [145]. The authors demonstrated that the ANA-Y response was reversible, and they tested it in fluorescence lifetime imaging (FLIM) mode, which established fluorescence lifetimes in both oxygen-free and oxygenated states (1.00 and 1.55 ns, respectively) [145]. ANA-Y and ANA-G were functional in a wide spectrum of medium acidities; however, maximum dynamic ranges were achieved at pH 8 [145]. Mutants with His348Ala substitutions are incapable of haem incorporation; therefore, they can serve as inactivated pH controls [145]. A ratiometric version of ANA-Y was also developed by fusing it with mCherry reference protein (ANA-Q), for which the signal is calculated as an emission ratio of 527 nm/602 nm (Figure 6C) [145]. 

In another report by Penjweini et al., the Myo-mCherry FRET probe was engineered, which consisted of mCherry as a fluorescence donor and myoglobin as a dark acceptor (Figure 6D) [146]. Upon oxygen binding, the optical properties of myoglobin change, which leads to the reduction of myoglobin absorption and mCherry emission spectra overlap. The red fluorescence of the chimeric protein, therefore, becomes enhanced in the presence of molecular oxygen due to the decreased efficiency of the energy transfer from mCherry to myoglobin. The Myo-mCherry probe was expressed in A549 cells and targeted to the mitochondria via a signaling sequence; its utility was proved when mitochondrial oxygenation states were visualized in the presence of different oxygen concentrations (from 20% to 0.5%) in two-photon FLIM mode [146]. 

Both ANA probes and Myo-mCherry represent a significant breakthrough in oxygen reporters’ development. As in the case of anaerobic GFP redding, they detect oxygen directly and their responses are reversible; but, more importantly, their mechanisms of functioning are better understood, which makes their performance more predictable and facilitates rational tuning. Moreover, it seems that they allow for real-time imaging with subcellular resolution; however, additional research on this issue is required. As their responses can be measured by FLIM, potential artifacts attributed to different expression rates or cellular motility are minimized. In addition, Myo-mCherry is based on a red FP, which is favorable for in vivo imaging, especially in the two-photon excitation mode. The need for an external cofactor is one of the issues that could hinder the implementation of these instruments under conditions where haem supply becomes limiting. Thus, in *E. coli* expression system, purified ANA-Y demonstrated a dynamic range only of 1.2-fold, which was improved either by the addition of 5-aminolevulinic acid (a precursor of haem biosynthesis) to the culture medium or by supplementation of the purified protein with hemin [145]. Another important question consists in the specificity of the probes related to the possible ability of the haem-containing part to interact with ROS or other compounds (for example, NO or CO). Finally, the differences in the biochemical properties of Venus and mCherry (such as a possible lag between maturation rates, temperature dependence, or pH dependence) might mimic the specific response of ANA-Q. 

### 2.2. Genetically Encoded Reporters of Redox and Metabolic Parameters

In this subsection, we will discuss the basic principles of GEFIs of some redox and metabolic parameters. The dynamics of these parameters and the most common representatives of these indicators are of interest in the study of hypoxia. In contrast to the oxygen/hypoxia reporter systems, these instruments have been extensively reviewed; therefore, we provide only the main ideas here. An extended list of biosensors, as well as some of their key parameters, can be found in the tables at the end of each section.

#### 2.2.1. Redox-Sensitive Fluorescent Proteins

With the development of hypoxia, both under physiological and pathological conditions, various protective mechanisms are activated in the cell. It is considered that hypoxia leads to the increased formation of ROS [147,148], primarily in the electron transport chain (ETC) in mitochondria as well as with the participation of NADPH oxidases [149]. In excess, ROS can damage the cell, as, for example, in hypoxia-reoxygenation injury [150]. On the other hand, smaller amounts of ROS can be involved in redox signaling, contributing to the initiation of transcriptional and post-transcriptional responses under conditions of oxygen deficiency. The most common ROS are the superoxide anion radical and hydrogen peroxide; hydrogen peroxide is formed from superoxide under the influence of the superoxide dismutase (SOD) enzyme. Hydrogen peroxide can oxidize protein thiol groups, resulting in changes of the redox states of individual cell compartments [149]. The change of the redox state leads to a change in the 2GSH/GSSG ratio and the reduced/oxidized thioredoxin ratio; because glutathione is present in high concentrations in cells (up to 10 mM), the oxidation state of the glutathione pool is a key parameter of redox equilibrium.

**Figure 7:**
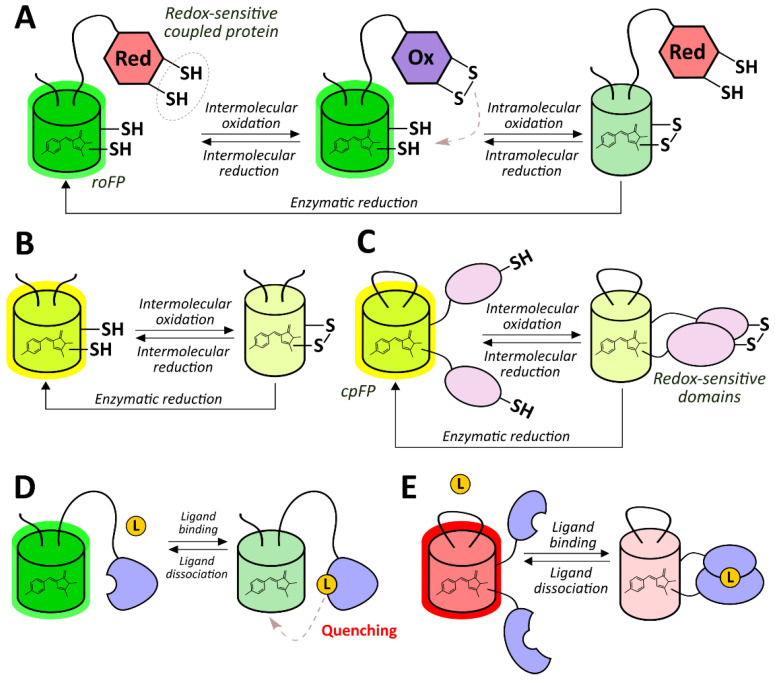
The main architectural types of genetically encoded fluorescent indicators (GEFIs) of metabolic and redox parameters based on a single optical reporter. Redox-sensitive fluorescent proteins (roFPs) with (**A**) or without (**B**) additional sensory domain. (**C**) Indicators based on a circularly permuted fluorescent protein (cpFP) with a sensory domain that can be modified by analyte. (**D**) Indicators based on the direct effect of a sensory domain-bound analyte on the chromophore of the FP. (**E**) Indicators based on the ligand binding by a sensory domain which transmits conformational changes to the chromophore microenvironment altering its optical properties.

To date, the developed redox-sensitive fluorescent proteins (roFPs) have made it possible to evaluate the redox states of glutathione pools in cells and in subcellular compartments (Table 1). The use of such indicators in the study of oxidative stress and redox signaling in hypoxia allows one to track the development of the cellular response from the first moments in real time. Currently, roFPs with sensor domains (Figure 7A) are primarily used, but their development began with simpler versions (Figure 7B). The basic principle behind simple roFPs is as follows: a pair of redox-active cysteines is introduced into an FP; under oxidation, a disulfide bond between the cysteines is formed, and the fluorescence intensity changes. The most popular indicators of this type are the roGFP probes [151,152]. These probes are ratiometric and have high response amplitudes and midpoint redox potentials, which are in the physiological range for redox-active cysteines. Later, the response rate of roGFP1 was improved, and a description of roGFP-Rx [153] probes was published. For use in a highly oxidizing environment, for example, in the endoplasmic reticulum, roGFP1-iX indicators with a reduced thermodynamic stability of the disulfide bond were developed [154].

The roGFP signal primarily depends on the redox state of the glutathione pool because the concentration of glutathione in the cell is very high. Nevertheless, several more specific sensors for glutathione have been developed involving a redox-sensitive FP that is fused to glutaredoxin, the enzyme that catalyzes the equilibration between GSH and GSSG (Figure 7A). Glutaredoxin fusion causes the indicator to show higher specificity to glutathione redox state and give a more rapid response. With this technique, Grx1-roGFP2 was constructed. This probe showed an approximately 100,000-fold faster response to GSSG compared to the original roGFP2 [155]. For more detailed information about roGFPs, see reviews [156,157]. The red-shifted redox FP, specific to glutathione, is also available today [158]. Grx1-roCherry had a design similar to Grx1-roGFP2: human Grx1 was fused to the redox-sensitive variant of red FP mCherry. Grx1-roCherry is more susceptible to oxidation than Grx1-roGFP2 due to an increased negative redox potential. This indicator was used in a hypoxia-reoxygenation model in parallel with Grx1-roGFP2-mito; it was shown that the cytosolic glutathione pool was much more stable compared to the mitochondrial pool when oxygen levels were changing.

Many redox-sensitive fluorescent probes whose signals rely on the influence of the redox status of the glutathione pool are currently available. However, a red indicator reflecting the redox state of the thioredoxin system was recently designed [159]. TrxRFP1 was constructed by engineering a redox relay between native redox-active human Trx1 cysteines and red roFP rxRFP1 [160] so that the fluorescence signal of rxRFP1 reflected the redox state of Trx1. The indicator was used with Grx1-roGFP2 to simultaneously monitor thioredoxin and glutathione redox state in live cells.

Most biosensors developed to date are based on GFP-like proteins. However, these proteins require oxygen for chromophore maturation; therefore, in conditions of hypoxia and anoxia, problems with sensor fluorescence may occur. A potential solution to this problem are sensors based on green fluorescent protein UnaG [115] from the Japanese eel. The protein’s fluorescence does not depend on oxygen; however, it requires a cofactor, bilirubin, to fluoresce. Redox-sensitive indicator roUnaG [161] was developed by introducing redox-active cysteines to the β-barrel surface of UnaG. This probe is intensiometric and displays a great dynamic range. 

#### 2.2.2. Genetically Encoded Fluorescent Indicators of ROS and RNS

A number of indicators that specifically report the concentration of one of the most important participants in redox metabolism, hydrogen peroxide, have been developed (Table 2). The HyPer family includes the largest number of sensors with different spectral characteristics. Several reviews are specifically devoted to HyPers [24,165]. The first indicator [166] was conceived as a chimera consisting of the regulatory domain of bacterial transcriptional factor OxyR and circularly permuted yellow fluorescent protein (cpYFP) integrated into the flexible region of OxyR-RD. Upon oxidation of key cysteine residues, OxyR undergoes a dramatic conformational rearrangement, and the fluorescence signal of cpYFP changes (Figure 7C). The original HyPer showed a ratiometric response and sensed submicromolar concentrations of H_2_O_2_. Descriptions of improved versions of HyPer [167,168] have also been published. A red-shifted variant, HyPerRed [169], is available today. A probe that is suitable for detecting H_2_O_2_ in highly oxidized conditions such as in the ER, called TriPer, is described in [170]. Due to the high pH-sensitivity of HyPer family probes, pH controls, HyPerX-C199S, are recommended to correct for pH effects during live-cell imaging. C199S mutants, otherwise called SypHers, have been used in research as pH-indicators [27,171,172]. Recently, HyPer7 [173], a significantly improved HyPer, was created. HyPer7 surpassed its predecessors in brightness, pH stability, and, most importantly, in sensitivity; the probe was at least 30-fold more sensitive than previous HyPers. Moreover, HyPer7 responded to H_2_O_2_ at least 60-fold faster than the original HyPer.

Several H_2_O_2_ reporters based on roGFP2 [174,175] have been developed. The most popular roGFP2-Orp1 [174] contains the yeast peroxiredoxin Orp1. Orp1 forms a redox relay with redox-sensitive roGFP2 that mediates roGFP2 oxidation by H_2_O_2_, causing a signal change. RoGFP2-Orp1 has slower response kinetics than HyPer, even though the sensitivity of two indicators is similar.

Although ROS play a key role in hypoxia, reactive nitrogen species (RNS), especially nitrogen oxide (NO•), signal hypoxia as well. NO• mimics oxygen and can reversibly inhibit isolated cytochrome c oxidase [176]. Additionally, the molecule can interact with the oxygen-sensing HIF pathway [177]. Various genetically encoded probes for NO• [178] were designed by fusing the GAF domain of transcription factor NorR, which contains a non-haem iron (II) center that binds NO•, with FPs of different colors, so that cyan, green, mint green, yellow, and orange indicators were obtained (Table 2). The geNOps mechanism of action is rather remarkable: the analyte impacts the chromophore structure directly due to the proximity to the chromophore of a conjugated FP (Figure 7D).

#### 2.2.3. Genetically Encoded Fluorescent Indicators of NAD(H) and NADP(H)

The effect of hypoxia on the redox status of a cell is associated with a change in metabolism. NADPH, as a key coenzyme in many biochemical processes, plays a role in maintaining the redox status of the cell, as glutathione is restored by NADP(H)-dependent glutathione reductase. On the other hand, NADP(H) is involved in the production of ROS from oxygen under the influence of NADPH oxidases. Therefore, the ratio of NADP^+^/NADPH together with the GSSG/2GSH ratio reflects the redox state of the cell. NAD(H), which is associated with NADP(H) through a reaction catalyzed by NAD^+^ kinase, plays an equally important role in biochemical processes. Because NAD(H) is a coenzyme of NAD(H)-dependent dehydrogenases that catalyze redox reactions in key metabolic processes such as glycolysis and the Krebs cycle, the NAD^+^/NADH ratio reflects metabolic changes and serves as a metabolic and redox indicator [179,180].

Today, NAD(H) fluorescent sensors are commonly used in research (Table 3), and most of them are based on bacterial redox-sensing repressor Rex, which undergoes pronounced conformational changes when the NAD^+^/NADH ratio is altered. As a fluorescent core, the biosensors contain circularly permuted fluorescent protein (cpFP), which is integrated into flexible regions of Rex or between two Rex subunits. Several reviews [181,182] are specifically devoted to the structure and properties of these indicators. Peredox [183], RexYFP [184], and SoNar [185] report NAD^+^/NADH levels. Of them, SoNar [185] has an advantage due to its ratiometric readout of a very high amplitude. The specificity of SoNar [185] was changed by rational mutagenesis, and iNap [186] sensors for the NADPH were obtained. In addition to the probes for NAD^+^/NADH ratio, there are probes that report only NADH (Frex and FrexH [187]) or only NAD^+^ [188] dynamics. Recently, Zou et al. presented a new highly responsive probe FiNad [189]. This indicator reflected the dynamics of NAD^+^/AXP ratio, where AXP was the total pool of ATP and ADP. However, because the total physiological pool of adenine nucleotides is usually maintained in homeostasis, authors suggested that the FiNad could actually be reported intracellular NAD^+^ fluctuations.

#### 2.2.4. Genetically Encoded Indicators of Metabolic Parameters

Continuing the discussion of the metabolic response to hypoxia, it is worth paying special attention to energy metabolism. In normoxia, ATP is primarily formed during oxidative phosphorylation in mitochondria. When the oxygen supply is limited, this metabolic pathway is inhibited, and ATP production via glycolysis is enhanced through the AMPK activation of several key glycolytic enzymes. The switch to glycolysis is not effective in long-term hypoxia, in which the adaptation of the cell by HIF activation occurs [148]. Chronic hypoxia is characterized by a decrease in oxygen consumption and a decrease in the intensity of ATP-utilizing processes [190]. Tracking the dynamics of ATP concentrations as well as the ATP/ADP ratio from the initiation of hypoxia became possible with the development of genetically encoded indicators.

There are a number of probes that can be used to record the dynamics of ATP concentration (Table 4). The structural foundation of most of the biosensors is the *ε*-subunit of the bacterial F_0_F_1_-ATP synthase. Several single FP-based biosensors of ATP have been developed. In ratiometric QUEENs [191], cpEGFP was integrated between two α-helices of the F_0_F_1_-ATP synthase ε-subunit. Upon ATP binding the ε-subunit undergoes conformational rearrangements and the signal of the fluorescent core changes (Figure 7E). After QUEENs were developed, the use of single-wavelength iATPSnFRs [192] indicators was reported. In these biosensors, the fluorescent core was replaced with circularly permuted superfolder GFP, which enabled improved folding in mammalian cells. Three intensiometric ATP sensors of different colors, MaLions, are described in [193]. In MaLions, the ε-subunit of the bacterial F_0_F_1_-ATP synthase was integrated into green FP Citrine, red FP mApple, and a variant of blue FP. These indicators allow for the simultaneous measurement of ATP in the cytoplasm and/or several organelles.

**Figure 8:**
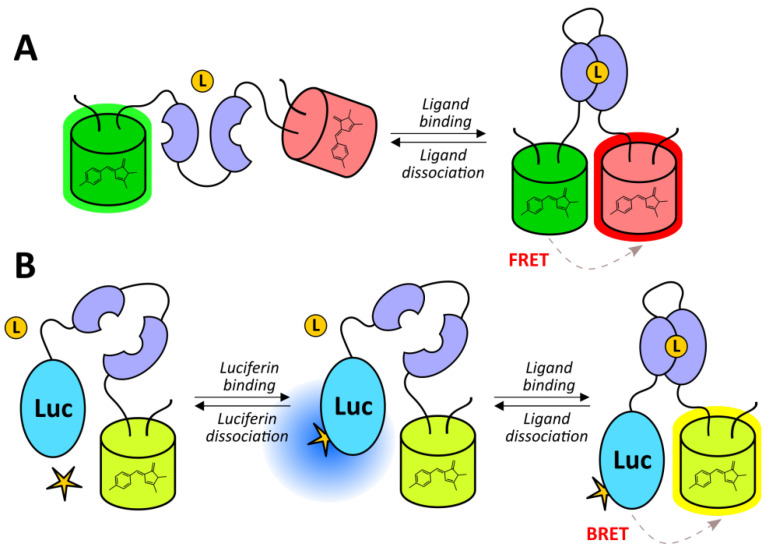
Genetically encoded indicators for metabolic and redox parameters based on two optical reporters. (**A**) FRET-based indicators with ligand-binding modules. (**B**) Bioluminescence resonance energy transfer (BRET)-based indicators with ligand-binding modules.

A group of FRET-based ATP indicators ATeams [194], and their red-shifted variants GO-ATeams [195] has also been developed (Figure 8A). GO-ATeams have several advantages over the original ATeams, such as increased pH-stability of the fluorescence signal and reduced phototoxicity of the exciting light. This group also includes BTeam [196] is a BRET-based biosensor that contains ATP-nonconsuming luciferase instead of a FRET-donor (Figure 8B). BTeam has a significant advantage over the previous ATP sensors: it does not require an external excitation light, so many of the common problems that are encountered when using FRET sensors are avoided.

Detecting changes in cellular ATP/ADP ratios is equally as interesting as measuring the dynamics of ATP concentrations in hypoxia studies. A probe called Perceval [197] was based on the bacterial protein GlnK1, which regulates ammonia transport associated with glutamine synthesis. This indicator could detect nucleotide ratios during extreme metabolic inhibition. An improved probe, PercevalHR, was developed [198]. Unlike the original Perceval, PercevalHR sensed more physiological nucleotide ratios.

The final product of glycolysis is pyruvate, which normally turns into acetyl-CoA, entering the tricarboxylic acid cycle. However, during hypoxia, pyruvate is converted to lactate by lactate dehydrogenase. Lactate accumulates in large quantities, spills out of hypoxic cells into circulation, and can be reused when the oxygen supply is restored [199]. The FRET probe called Laconic [200] was developed to detect lactate dynamics (Table 4). This indicator has been used in vivo to study lactate metabolism in mouse brain cells in real time [201].

#### 2.2.5. Genetically Encoded pH Indicators

Hypoxia is accompanied by a decrease in intracellular pH. To date, a multitude of fluorescent indicators that reflect the dynamics of intracellular pH have been developed [202]. Red indicators are often preferred due to less phototoxicity and better tissue penetration of the excitation and emission light. Additionally, they allow for multiparameter imaging, as they can be used in parallel with green indicators (Table 5). Of the red pH sensors, pHoran4 and pHuji [203], pHTomato [204], pHRed [205], and mNectarine [206] are worth mentioning. Each probe displays a good dynamic range (~3 for pHTomato and >10 for the others); in addition, pHRed gives a ratiometric response, which is a rare phenomenon for red biosensors. Interestingly, pHRed was designed based on long Stokes FP mKeima with a Stokes shift of approximately 180 nm [207]. In contrast, of the green and yellow probes, ratiometric indicators are much more common due to the ability of the GFP chromophore to protonate. For example, ratio-pHluorin [208], pHluorin2 [209], dual-emission probes deGFPs [210], EYFP [211], and E^2^GFP [212] are GFP mutants, and they do not include any other sensory domain. The exception is ratiometric SypHer3s [172], an improved pH control variant of the hydrogen peroxide indicator HyPer [166].

## 3. In Vitro Models for Real-Time Imaging of Cell Signaling and Metabolism during the Course of Hypoxia

In vitro models, such as neuronal and retinal cell cultures, brain slice systems [214,215] and retina organ cultures [216], used to investigate the pathogenesis of hypoxic damage or for drug discovery applications are appreciated primarily because they provide high levels of reproducibility due to well controlled culture and treatment conditions. These models are also amenable to a variety of advanced techniques for live imaging (fluorescence, confocal, and multiphoton microscopy and state-of-the-art microscopic techniques such as FLIM, total internal reflection fluorescence (TIRF), FRET, super resolution, etc.) and electrophysiological recordings. The ability to combine various assays in one sample and the availability of a plethora of genetically encoded and chemogenic indicators for probing metabolites and signaling molecules allows for the multiparametric monitoring of physiological processes that occur during the course of hypoxia exposure in in vitro models. At the same time, the absence of laborious surgical manipulations for modeling hypoxic/ischemic damage in vitro simplifies procedures and reduces any dependence on the experimenter’s skill. These models also allow for the study of the pathogenesis of hypoxic damage in different genetic backgrounds [217,218]. This is very important for the determination of genetic mechanisms that underlie variable vulnerabilities to hypoxia [219,220].

To induce hypoxia in vitro, two types of systems are commonly used. One system consists of a hypoxic chamber that is connected to a gas mixing station or a tank containing a pre-mixed gas. To create a hypoxic environment, a gas mixture of the desired partial pressure of O_2_ is pumped over a chamber. The level of hypoxia can be adjusted by varying the O_2_ content in the gas mixture. Another system consists of a flow-through chamber connected to a special station in which the culture medium is equilibrated in an atmosphere with the desired O_2_ content and then pumped over the flow-through chamber to create a hypoxic environment. The use of a hypoxic chamber or a flow-through chamber provides great flexibility in choosing specific hypoxic conditions. Various types of hypoxic and flow-through chambers for specific experimental conditions have been described, and many of them have been commercialized. To mimic the processes underlying a stroke, cell cultures or brain slices undergo oxygen-glucose deprivation followed by subsequent reperfusion. This is a broadly accepted in vitro model of stroke. In addition to hypoxic and flow-through chambers, diverse microfluidic platforms have been developed to create well-controlled hypoxic environments in in vitro systems [221,222,223]. 

Neuronal and retinal cell cultures from various sources represent the most convenient models for studying hypoxic injury of the CNS. In addition to the benefits of in vitro models that were previously mentioned, they offer additional advantages. The resolution of cell analysis may be enhanced by using advanced cell biology techniques such as flow cytometry, various types of cell sorting, single-cell analysis, microfluidics, etc. [221,224,225,226,227]. Automation of most live cell imaging platforms and cell manipulation techniques enables a variety of high content screenings that provide information about the physiological processes that occur during the course of hypoxia. This is very useful not only for studying the pathogenesis of hypoxic damage but also for drug discovery applications. Cultured cells can easily undergo genetic manipulations, allowing for the multiparametric live cell imaging of cell signaling and metabolism during the course of hypoxia. Moreover, innovative genetic tools for opto- [228], chemo- [229,230], and thermo-genetics [231,232] can be easily delivered into cultured cells to govern their physiological activity, thus modulating the cell response to hypoxia. Finally, combining technologies for the generation of adherent neuronal cell cultures and cerebral organoids from ESCs or iPSCs with state-of-the-art genome editing methods based on CRISPR/Cas9 offers innovative avenues for studying the genetic mechanisms of neuronal vulnerability to hypoxia in human cells. Similar approaches have been successfully tested in some models of neurodegenerative disorders [233,234,235,236].

Cerebral organoids derived from human ESCs or iPSCs are currently considered to be the most promising models for studying many aspects of human brain development and the pathogenesis of various neurological and psychiatric disorders [237,238]. They can also be useful for investigating hypoxia-induced pathogenesis and for drug discovery applications. Cerebral organoids simulate the architecture and functioning of the human embryonic brain and, therefore, may serve as a platform to model neonatal hypoxia-ischemia and reoxygenation. Recently, the effects of oxygen deprivation on corticogenesis in vitro were evaluated in cortical spheroids derived from human iPSCs of healthy patients or human ESCs (line H9) [239,240]. Generated human cortical spheroids have been found to simulate the hallmarks of the mid-gestation human cortex. Therefore, they could be used to model hypoxic encephalopathy induced by second trimester placental insufficiency. Studies using these types of models have shown distinctions in the susceptibility of different neuroprogenitor subtypes to hypoxic injury. These observations may explain the cellular mechanisms underlying specific pathogenetic patterns in the developing human brain affected by hypoxia. However, the reported experimental platforms lack immune cells and a vascular system; therefore, they are incapable of simulating the inflammatory response that is known to accompany hypoxic damage. The most recent study has reported the fabrication of vascularized cerebral organoids from human ESCs [241]. These cerebral organoids exhibited several hallmarks of blood-brain barrier formation. Although this is still far from a fully functional blood-brain barrier, the advent of this technology offers unmatched opportunities for modeling hypoxic injury of the human brain in vitro.

Brain slice systems and organ cultures are widely used as in vitro models for studying normal brain or retinal functioning as well as the pathogeneses of various injuries and diverse neurodegenerative processes [216,242]. Slice systems can be created from a variety of brain regions such as the hippocampus, cortex, cerebellum, striatum, or spinal cord [242,243]. Retinas are usually maintained as organ cultures [216]. Slices and organ cultures possess some advantages over both cell culture-based models and animal models. Unlike cell culture-based models, brain slices and organ cultures largely preserve cellular composition, 3D structure, and neuronal connections of the original brain or retina tissue. Neuronal activity, synaptic transmission, astrocyte and oligodendrocyte functioning, microglial response, components of the blood brain barrier, and many other aspects of cellular interactions have been shown to be maintained for prolonged time intervals in brain slices and retinal organ cultures [243,244,245,246,247]. Thus, slice systems and retinal organ cultures replicate many aspects of the in vivo situation. At the same time, slices and organ cultures have many advantages of the in vitro systems, such as creating well-controlled culture and treatment conditions for modeling nervous tissue injury and for drug discovery applications, availability for non-viral genetic manipulations [248,249], and accessibility for multiparametric monitoring by using live imaging and electrophysiological recording. 

Hypoxia alters free radical formation and shifts redox regulation in cells [135]. Therefore, numerous studies in the field have exploited genetically encoded redox indicators to dynamically visualize redox signaling in in vitro models during the course of hypoxic exposure. For instance, the simultaneous expression of Grx1-roCherry in cytoplasm and Grx1-roGFP2 [155] in mitochondria allowed for the observation of redox state dynamics of the glutathione pool in distinct compartments of HeLa cells during the course of hypoxia/reperfusion exposure [158]. The mitochondrial 2GSH/GSSG ratio was found to decrease after hypoxia onset, and it was followed by recovery during reperfusion, while the redox state of the cytoplasmic glutathione pool remained unaffected. This observation confirms the primary role of mitochondria in producing ROS during hypoxia/reperfusion exposure.

Exposure to hypoxia has been shown to readjust circadian rhythms of cellular metabolism in zebrafish embryonic fibroblasts (Z3) [250]. The circadian fluctuations of cytosolic and mitochondrial H_2_O_2_ and NAD^+^/NADH redox couple were measured by recording fluorescence signals from the roGFP2-Orp1 [174] and Peredox-mCherry [183] sensors, respectively. It was found that exposure to hypoxia decreased mitochondrial H_2_O_2_ levels. In addition, the rhythms and amplitudes of circadian oscillations of cytosolic H_2_O_2_ remained unchanged in hypoxia-treated cells. Although it remained constant under normoxic conditions during the entire circadian cycle, the NAD^+^/NADH ratio began to oscillate after hypoxia exposure. These results, in addition to other findings of this study, hint at the mechanisms of how metabolic shifts induced by hypoxia may readjust cellular circadian clocks.

Variability in oxygen levels has been shown to coordinate multiple physiological, pathological, and developmental processes in multicellular organisms [251,252,253,254]. For instance, tumor cells are thought to migrate toward blood vessels, guided by local oxygen gradients [255]. Recently, in an original in vitro model, researchers created hypoxic conditions via the confinement of immortalized mammary gland MCF10A cells in a small volume of culture medium; they found that the directed migration of cells toward oxygen was associated with the occurrence of an H_2_O_2_ gradient at the margin of a cell cluster [256]. Occurrence of the gradient was observed by the real-time recording of fluorescence signals of co-expressed HyPer-3 [168] with H_2_O_2_-insensitive HyPerRed-C199S [169] to monitor pH changes in the system. Increased H_2_O_2_ levels at the margins of cell clusters have been shown to potentiate the activity of EGF receptors, thus guiding cell migration from regions of low to high oxygen. Similarly, pH gradients were observed in cell spheroids generated from HeLa cells stably expressing the pH sensor SypHer2 [27]. Live imaging revealed lower intracellular pH values inside the cores versus the peripheral regions of HeLa cell spheroids. Observed pH gradients have been suggested to appear due to a hypoxia-induced shift in cell metabolism in the inner regions of HeLa cell spheroids.

Discovering novel drugs for preventing, reversing, or slowing the progression of the negative consequences of hypoxic injury is a high priority in the field. The combination of in vitro models with the real-time imaging of cell signaling molecules and metabolites by genetically encoded biosensors is currently the most powerful tool for the multiparametric screening of novel and existing drugs. Such an approach could enable the identification of a promising drug for subsequent testing in pre-clinical trials. Recently, the genetically encoded ATP sensor ATeam1.03 was used to screen drugs that maintained ATP levels in the mitochondria and cytosol of cultured cardiomyocytes under hypoxic conditions [257]. This study identified ivermectin as a drug that not only maintained ATP levels under hypoxia exposure but also exhibited an anti-hypertrophic cardioprotective effect. Several studies have also reported the successful application of a variety of genetically encoded biosensors for recording cellular redox signaling responses to hypoxia exposure in plant and bacterial systems [161,258,259].

**Figure 9:**
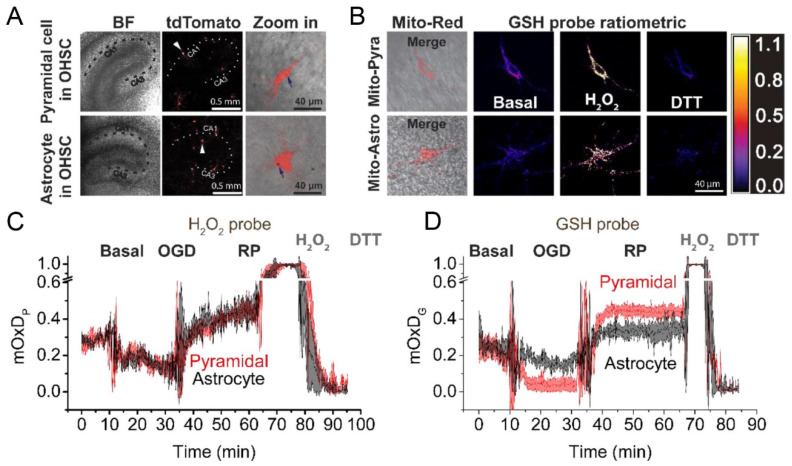
Pyramidal and astrocyte mitochondrial H_2_O_2_ respond similarly and GSH systems respond differently to oxygen-glucose deprivation and reperfusion. (**A**) Demonstration of the expression of tdTomato (Ex: 561 nm, Em: 580–600 nm) fluorescent protein in a pyramidal cell (top) and an astrocyte (bottom) from organotypic hippocampal slice cultures (OHSCs) (gene gun). (Left) bright field (BF) image of the OHSC and (middle) fluorescence image of tdTomato are taken with a 5× objective lens. The dotted line indicates approximately the *Cornu Ammonis* (CA). (Right) Enlarged images of single cells (indicated by arrows in the middle image) are taken with a 63× objective lens. Gold particles (see blue arrow) carrying plasmids were introduced to the cell by gene gun. (**B**) Representative images of a pyramidal cell (top) and an astrocyte (bottom) expressing mitochondrially targeted fluorescent protein. (Left) overlay of BF image and fluorescence image of and Mito-Red (Ex: 561 nm, Em: 580–600 nm). (Middle/right) ratiometric images of the mitochondrial GSH probe (Mito-GP, Ex: 405/488 nm, Em: 500–530 nm) at basal, H_2_O_2_, and dithiothreitol (DTT) treatment. (**C**,**D**) The oxidation degree (OxD) derived from the fluorescence measurements during oxygen-glucose deprivation and reperfusion. OHSCs were sequentially treated with 10 min basal/20 min oxygen-glucose deprivation/30 min reperfusion followed by H_2_O_2_ and DTT sequentially for calibration. Reprinted bypermission from American Chemical Society: ACS Chemical Neuroscience [278], copyright 2018.

Primary neuronal or retinal cell cultures, ESC- or iPSC-derived neuronal or retinal cells, ESC- or iPSC-derived cerebral organoids, brain slices, and retinal organ cultures are widely used to study a variety of questions concerning cell signaling, cell communication, and metabolism in the CNS. Numerous studies have reported the successful application of genetically encoded biosensors that are sensitive to a variety of metabolites and signaling molecules, including ions and neurotransmitters, to investigate the functioning of neurons and glia in in vitro systems under diverse physiological (spontaneous and evoked activity of neuronal and glial cells) or pathological (Parkinson’s disease, Rett syndrome, and spinal muscular atrophy) conditions. Genetically encoded biosensors were applied to visualize the release of neurotransmitters and their intracellular dynamics [260,261,262], to observe the metabolic activity (glucose, pyruvate, ATP) and functioning of second messenger systems (Ca^2+^, cAMP) [263,264,265,266], to detect the formation of ROS [267,268,269,270], and to record the responses of cellular redox systems [183,271,272,273,274,275]. However, many fewer studies have attempted to exploit genetically encoded sensors for the real-time evaluation of alterations in cell signaling and metabolism in in vitro models of brain hypoxic injury. For instance, the responses of the glutathione redox system in neuronal cytoplasm and mitochondria during the course of oxygen-glucose deprivation and reperfusion in organotypic brain slices (an in vitro model of the stroke) were recorded by using the genetically encoded fusion biosensor Grx1-roGFP2 [276]. Under precisely controlled treatment conditions within a sample, the fluorescence signal from the biosensor was accurately calibrated using a signal from the co-transfected fluorescent protein tdTomato as an internal standard. This enabled not only the detection of perturbations of the redox couple GSH/GSSG but also the conversion of the fluorescence signal into quantitative measurements of the oxidation degree of the sensor. The latter allowed for the quantitative determination of the reduction potential of the glutathione system in the neuronal cytoplasm and mitochondria during the course of oxygen-glucose deprivation and reperfusion. Using their improved protocol, the authors were able to define time dependence of the redox state of the glutathione system in this in vitro model of the stroke [276]. Moreover, they demonstrated that the mitochondrial reduction potential became more reducing during oxygen-glucose deprivation and more oxidizing during reperfusion, while the cytoplasmic reduction potential did not change significantly. In their other studies, the authors applied genetically encoded fluorescent biosensors roGFP2-Orp1 [174] and Grx1-roGFP2 [155] to track and compare the dynamic changes in hydrogen peroxide levels and the oxidation state of the glutathione redox system in the mitochondria of single CA1 and CA3 pyramidal cells [277] as well as single CA1 pyramidal cells and astrocytes [278] using a model of oxygen-glucose deprivation and reperfusion in organotypic hippocampal slices. Comparing the patterns of dynamic changes in hydrogen peroxide levels and oxidation states of the glutathione redox system during distinct phases of oxygen-glucose deprivation and reperfusion revealed differences in the tuning of redox metabolism in all studied cell types. For instance, the mitochondria of CA1 pyramidal cells had a more oxidizing environment than the mitochondria of CA3 pyramidal cells during the reperfusion phase [277]. Likewise, the oxidation state of the glutathione redox system was found to change more in CA1 neurons than in astrocytes, while hydrogen peroxide levels in both cell types remained stable during all phases of oxygen-glucose deprivation and reperfusion [278] (Figure 9). These findings have elucidated the differences in vulnerability to hypoxic injury between CA1 and CA3 pyramidal cell subtypes and astrocytes as well as provided novel insights into the mechanisms by which astrocytes secure neurons.

**Figure 10:**
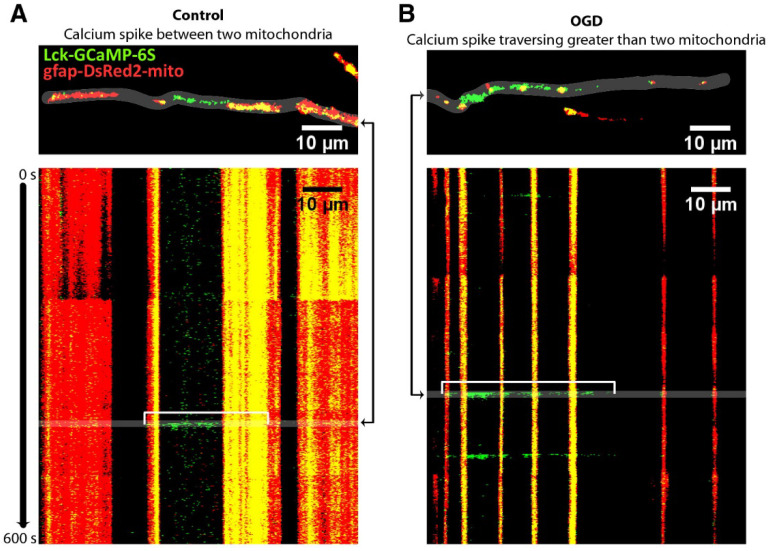
Extramitochondrial Ca^2+^ spikes were no longer contained between two mitochondria after oxygen-glucose deprivation. (**A**) Representative image of a single frame (top) displaying an extramitochondrial Ca^2+^ spike (Lck-GCaMP-6S, green) contained between two mitochondria (gfap-DsRed2-mito, red), and a kymograph (bottom) displaying Ca^2+^ and mitochondria over time throughout the recording acquired 24 h after control treatment, with the example spike indicated by a white bracket. (**B**) Representative image of a single frame (top) displaying an extramitochondrial Ca^2+^ spike (green) traversing >2 mitochondria (red), and a kymograph (bottom) displaying Ca^2+^ and mitochondria over time throughout the recording acquired 24 h after oxygen-glucose deprivation, with the example spike indicated by a white bracket. The times at which the representative frames appear in the kymographs are indicated by the black arrows to the side of the image [279].

Several reports have described the role of hypoxia-induced intracellular calcium elevations in the pathogenesis of hypoxic damage. For instance, calcium activity in astrocytes and the relationship of this activity to the loss of mitochondria residing in astrocyte processes were examined in a model of oxygen-glucose deprivation and reperfusion in organotypic brain slices [279]. Slices were co-transfected with two constructs under the control of the minimal glial fibrillary acid protein (GFAP) promoter: the genetically encoded calcium sensor GCaMP6s [280] for expression in the cytoplasm and the fluorescent reporter dsRed targeted to the mitochondria. Morphological evaluation showed a fragmentation and an autophagic degradation of the mitochondria in astrocyte processes after transient oxygen-glucose deprivation. Live imaging performed at 24 h after oxygen-glucose deprivation revealed the following when compared to control samples: a 2-fold increase in the amplitude as well as an altered spatial spread of spontaneous calcium elevations in the cytoplasm surrounding the mitochondria and in the spaces between the mitochondria in astrocyte processes (Figure 10). These observations indicate that the loss of mitochondria in astrocyte processes increases astrocyte calcium signaling after oxygen-glucose deprivation, thus affecting the functioning and survival of astrocytes.

Similarly, the role of cytosolic and mitochondrial calcium elevation in the pathogenesis of hypoxic injury has been studied in a model of oxygen-glucose deprivation and reperfusion using the PC12 cell line with the genetically encoded calcium indicator D3cpv [281] and its mitochondria-targeted variant, 4mtD3cpv [282]. In this study, the modulation of calcium T-type channels Ca_v_31 and Ca_v_3.2 via inhibition with Ni^2+^, siRNA-mediated Ca_v_3.1/Ca_v_3.2 silencing, or the overexpression of Ca_v_3.2 was combined with real-time ratiometric imaging of calcium activity in the cytosol and mitochondria of PC12 cells during the course of hypoxia exposure combined with a subsequent cytotoxicity assay at the reperfusion phase. The inhibition or silencing of T-type channels was found to change the kinetics of cytosolic calcium elevation, which led to delayed ATP decline (recorded by ratiometric imaging of FRET-based ATP indicator ATeam [194]) during the course of oxygen-glucose deprivation and to reduced cell death at the reperfusion phase. In contrast, the overexpression of Ca_v_3.2 delayed the elevation of cytosolic calcium, which increased cytotoxicity. Cytosolic calcium increases were accompanied by mitochondrial calcium increases. Modulating the expression of the major calcium uptake protein of mitochondria via overexpression or shRNA-mediated silencing revealed that the cytotoxicity evoked by the hypoxia-induced calcium influx into PC12 cells was primarily mediated by mitochondrial calcium overload rather than an elevation in cytosolic calcium. These observations indicate that the calcium transfer from the cytosol to the mitochondria via T-type calcium channels seems to cause increased cytotoxicity in PC12 cells [282].

Another study examined the contribution of brainstem hypoxia to the pathogenesis of systemic arterial hypertension in spontaneously hypertensive rats [283]. In this study, organotypic brainstem slices were exposed to transient hypoxia, and the calcium responses of sympathoexcitatory neurons residing in the C1 area of the rostral ventrolateral medulla were detected by ratiometric imaging using the genetically encoded calcium indicator TN-XXL [284]. Sympathoexcitatory C1 neurons play an important role in the regulation of sympathetic vasomotor tone, and their activation increases arterial blood pressure. The neurons are also known to be sensitive to hypoxia. It has been shown that transient hypoxia in organotypic brainstem slices evokes the release of ATP and lactate and increases calcium activity in sympathoexcitatory C1 neurons. In addition, the inhibition of ATP- and lactate-mediated signaling pathways has been found to markedly reduce hypoxia-induced calcium elevations, indicating that ATP and lactate mediate the excitation of sympathoexcitatory C1 neurons under hypoxic conditions [283]. These observations shed light on the mechanism by which brainstem hypoxia contributes to high systemic arterial blood pressure in spontaneously hypertensive rats.

The aforementioned reports demonstrate how the illumination of cell signaling and metabolic activities with genetically encoded biosensors provides novel information about the pathogenesis of hypoxic injury in in vitro systems. However, although they are convenient models of brain hypoxia, in vitro systems lack many important aspects of brain hypoxic injury. For instance, these models, including live slices and vascularized cerebral organoids, poorly recapitulate blood-brain barrier functioning. Therefore, they largely fail to replicate complex vascular and inflammatory responses that accompany brain hypoxic/ischemic injury in vivo. These responses are known to govern delayed postinjury processes. Furthermore, in in vitro models, evaluating neurological deficits evoked by brain hypoxic/ischemic damage is not feasible. This limits the predictive power of these models for drug discovery applications. Creating primary cell cultures and slices is generally associated with manipulations and transient perturbations in nutrient and oxygen supplies. This can change cell signaling and metabolism, potentially affecting the picture of subsequent hypoxic injury. These drawbacks of in vitro models should be considered when contemplating the use of live imaging with genetically encoded biosensors in an experiment.

## 4. Animal Models for Real-Time Imaging of Cell Signaling and Metabolism during the Course of Hypoxia

Although complicated to use, an organism is the most reliable model system for replicating diverse human pathophysiologic processes. This is primarily because many researchers frequently face the following problem: an exciting phenomenon or an encouraging drug effect that has been found in an in vitro system is poorly reproduced or even fails in an animal model. Unfortunately, no in vitro model is able to completely replicate the complex interorgan, intercellular, and molecular interactions that normally occur in an intact organism. Many physiological systems, for instance, immunity and blood circulation, respond to hypoxic/ischemic injury of the brain, and their functioning determines the vulnerability of the brain to hypoxic/ischemic injury. Moreover, the pathogenesis of hypoxic/ischemic injury is underscored by a non-uniformity of brain tissue damage, and this feature is difficult to properly recapitulate in in vitro model systems. Therefore, animal models are designed to address questions regarding the pathogenetic mechanisms of brain hypoxic/ischemic injury in the context of complex physiological interactions.

Hypoxia underlies the pathophysiology of common and dangerous diseases of the nervous system such as stroke and hypoxic-ischemic encephalopathy (HIE) [285,286,287]. Hypoxia-induced neurodegeneration is connected to sleep apnea [288] and acute or chronic disorders of spinal cord blood supply [289]; it is linked to Alzheimer’s disease [290], amyotrophic lateral sclerosis (ALS), and spinal muscular atrophy (SMA) [289]. Many animal models are available for these pathologies, and some are quite compatible with the use of genetically encoded biosensors. However, to our surprise, examples of such research in the literature are very rare. Most studies analyze end points (such as stroke volume, gene expression levels, or biochemical parameters) without attempting to track the progress of events in real time. In this part of the review, we will list experimental approaches that can be employed for in vivo studies of hypoxia-induced processes in the nervous system using genetically encoded biosensors and describe several inspiring examples of such research in other fields.

The effects of hypoxia on the nervous system have been studied in different animals, and each model has advantages and disadvantages. Among vertebrates, *Xenopus* and zebrafish embryos and rodents seem to be the most convenient for using biosensors in vivo. Young zebrafish larvae and *Xenopus* tadpoles are transparent, which allows for the direct noninvasive observation of fluorescent signals from inside living animals. For studying highly pigmented tissue with low optical accessibility (e.g., retina), special mutant fish strains are available [291]. The techniques for generating transgenic zebrafish are well established, and a vast number of genetically modified zebrafish lines exist [292], including lines expressing genetically encoded sensors targeted to the CNS [260,261,293,294,295,296,297]. When a ready-to-use line is not available, the methods for quickly generating zebrafish larvae transiently expressing the sensor of interest can be utilized [298]. All of the above (except the availability of pigmentless mutants) are true for *Xenopus* tadpoles as well [299].

The small size and transparency of zebrafish and *Xenopus* larvae allow the use of virtually any type of fluorescence microscopy for GEFI signal detection in vivo, depending on the particular task. Before examination, a larva is normally lightly anesthetized by Tricaine and immobilized in a drop of low-melt agarose in a glass-bottom Petri dish [300]. Such preparations have been successfully employed in many studies involving biosensors. Hypoxic conditions can be created, for example, by placing a Petri dish with a larva on a microscope stage in an incubator with oxygen control [301] or by the perfusion of deoxygenated water through a sealed chamber containing the fish. The latter approach combined with the implementation of a microfluidics system was proven to be efficient for switching between different oxygen concentrations in the medium around the larvae within seconds [302]; this method looks promising for the modeling of ischemia/reperfusion conditions.

One of the most exciting examples using genetically encoded biosensors in zebrafish and *Xenopus* was a series of studies utilizing the hydrogen peroxide indicator HyPer, which revealed the central role of H_2_O_2_ signaling in development, regeneration, and inflammatory responses ([303,304,305,306,307,308,309,310,311], reviewed in [23,312]). The first generation of HyPer, whose H_2_O_2_ detection capabilities made all these findings possible, has one drawback in common with many other FP-based biosensors—it is pH-sensitive. It is necessary to use HyPer with the specially designed pH-control, SypHer [171], which complicates experiments. In hypoxia research, the pH sensitivity of the probe can be particularly limiting because the pH of oxygen-deprived tissues typically decreases [313,314,315]. The recent development of pH-independent and more sensitive probe HyPer-7 will make such experiments more robust and applicable for in vivo hypoxia studies [173].

Another striking example of opportunities provided by in vivo imaging with genetically encoded sensors is the use of calcium indicators of the GCaMP [316] family for studying the brain circuitry of zebrafish larvae. Advances in imaging techniques, especially in one-photon [317,318] and two-photon [319] light sheet microscopy, together with the development of the optimized GCaMP variants [280,320,321], have enabled previously unattainable recordings of whole brain activity in the vertebrate animal with single-cell resolution. Further technical improvements allowed for the application of whole-brain neuronal activity imaging in freely swimming zebrafish larvae [322,323,324,325], making it possible to study the neuronal basis of the whole behavioral repertoire of this animal. 

The emergence of this exciting array of tools will undoubtedly revolutionize the field, especially considering that these methods are compatible with optogenetic approaches to control the activity of neurons [326]. It is already possible to describe the neuronal ensembles involved in motor adaption [327], navigation in the virtual environment [328], visual perception and prey detection [321,329], exploratory locomotion [330], vestibular stimulation [331], transition from active to passive behavior in response to stress [332], etc. The same approaches were successfully employed for the brain-wide monitoring of pathological neuronal activity during chemically-induced seizures [294,333,334,335] and in a high-throughput functional connectivity analysis platform for drug screening [336]. 

The above-mentioned studies illustrate the great potential of the use of genetically encoded biosensors in living zebrafish. However, this approach is rarely applied in hypoxia studies. One of the few (if not the only) examples of such research is a recent study by Kioka et al., where they studied the protective role of the hypoxia-inducible protein G0/G1 switch gene 2 (G0s2) against hypoxia in beating zebrafish hearts. Using the mitochondria-targeted FRET-based ATP biosensor ATeam [194] in zebrafish loss-of-function and gain-of-function g0s2 models, Kioka et al. demonstrated that G0s2 enhanced ATP production in the mitochondria of cardiomyocytes in a cell-autonomous manner, protecting heart function under hypoxic conditions [301]. The authors hypothesized that G0s2 is involved in the mechanism of preconditioning-induced ischemic tolerance, a phenomenon widely known to occur in brain tissue as well [337]. It would be interesting to investigate whether G0s2 enhances ATP production in neurons or has similar neuroprotective effects against ischemic nervous system damage.

Despite the advantages provided by transparency and the simplicity of genetic manipulations of zebrafish and *Xenopus* larvae, mice and rats explicably remain far more popular models of human diseases. Many rodent models of hypoxic brain damage are available. The most commonly used ischemia models include unilateral common carotid artery ligation (the Rice–Vannucci model) [338] and middle cerebral artery occlusion (MCAO) [339]. Both models are adapted for mice and rats of different ages [340,341]. Cerebral ischemia can also be mimicked in embolic models by injection of autologous blood clots or artificial materials [342]. Thus, in models of small vessel disease, occlusion can be created via the injection of cholesterol-derived microcrystals and fluorescent microspheres [343] as well as by the local activation of photosensitizing dye injected into the bloodstream [344,345]. The above models are used to create focal damage. Cerebral hypoperfusion can be obtained by the constriction of vessels by local endotelin-1 injections [346] or by artery stenosis [347]. The latter, along with cardiac arrest techniques, can be used to achieve global hypoxia [348]. A detailed description of these and other models with their advantages and disadvantages is presented in numerous reviews [286,340,341,342,348,349,350,351,352]. Described hypoxia models seem compatible with brain imaging using GEFIs.

The development of CRISPR/Cas9 genome editing technologies significantly improved and simplified the development of new transgenic mouse lines [353,354,355]. Many lines expressing GEFIs in the brain already exist [28,30,356,357,358,359,360,361,362]; in addition, a number of lines express GEFIs in a Cre recombinase-dependent manner and can be bred into distinct Cre recombinase driver mice to direct expression of sensors in the selected cell populations [363]. Some transgenic lines are commercially available (jax.org). For transient expression, in utero electroporation [364] and injections of recombinant adeno-associated viruses or lentiviruses into the region of interest in the brain can be used for gene delivery [365,366]. 

Several approaches can be applied to visualize GEFIs in the brain. A cranial window, open or thinned, is implanted when visualizing the cortical surface and subcortical structures [367]. When cellular resolution is not the goal, wide-field mesoscopic imaging of large regions of the brain surface can be performed [368]. For cellular resolution, the use of two-photon microscopy is preferable [369]. Typically, two-photon microscopy obtains images at a depth of up to 900 microns, but it is possible to perform calcium imaging in the hippocampus at a depth of up to 1200 microns [370]. The use of three-photon microscopy allows for imaging at even greater depths [371]. 

Imaging of deep structures can be performed by the implantation of an optical fiber or an endoscopic lens. The first is suitable for studying the dynamic activity of brain regions because it does not have sufficient resolution to visualize individual cells [372]. The use of endoscopic lenses in combination with miniature microscopes (miniscopes) offers incredible opportunities for imaging. Only previously available for fixed head experiments, two-photon microscopy coupled with miniature or even wireless microscopes [373] allows the visualization of processes in the brain of actively moving animals in a wide range of behavioral tests [374].

The first studies utilizing genetically encoded markers for in vivo imaging of events taking place during brain ischemia were performed on transgenic C57Bl/6 mice expressing green and yellow fluorescent protein within a subset of neurons [375]. Murphy and colleagues, along with other groups, used two-photon microscopy through cranial windows to track acute and long-term changes in neuronal morphology in real time using different ischemia models [376,377,378,379,380,381,382,383]. Of the many observations made in this set of experiments, Zhang et al. showed [377] that the degree of dendrite blebbing (the stereotypical pattern of dendrite degeneration, expressed by segmental dendritic beading and loss of spines [384,385]) depended greatly on the severity of the ischemia. While severe ischemia (<10% of blood supply), modeled by local photothrombosis, led to the widespread loss of dendrite structure within minutes, the less effective (~50% blood supply) endotelin-1-induced ischemia did not significantly reduce the number of spines during the first 5 h after onset. At longer time points (>7 h), even moderate ischemia resulted in spine loss. However, the most exciting finding was that if reperfusion occurred within 20–60 min after photothrombosis, the dendrite structures and spines partially recovered [377,381]. Further studies using the same approaches defined the detailed spatio-temporal relationships between blood flow restrictions and dendrite damage [379,380,381].

Neuronal mitochondria appear to be more sensitive to ischemia than the dendrites themselves. In other experiments using transgenic mice expressing mitochondria-targeted cyan FP in neurons [386,387], it was demonstrated that mitochondrial fragmentation began to develop during the first 5 min of ischemia. Fast reperfusion allowed the recovery of mitochondria structures; however, only 8 min of occlusion led to irreversible fragmentation. Overall, the mitochondrial response to the ischemic damage developed much faster than dendritic beading, and in contrast to the latter spread beyond the ischemic core with time [387].

GFP-expressing transgenic mice were also used to investigate the connections between stroke-induced spreading depolarization (SD), dendritic beading, and astrocyte swelling [388,389,390]. It was demonstrated that transient blebbing of dendrites strongly correlated with SDs and that the probability of dendritic recovery was linked with the presence of an uninterrupted blood supply nearby [388]. Astroglial swelling was either transient or permanent, depending on the duration of the depolarization wave and the severity of ischemia [390].

Labeling brain cells with FPs allows for real time tracking of their morphology and viability. The use of genetically encoded biosensors, which is not much more technically complex, provides far greater opportunities for studying hypoxia-induced intracellular processes in vivo. However, to date, there are only several proof-of-principle studies utilizing this approach, and they only employ Ca^2+^ sensors of the GCaMP family. Murphy and colleagues have developed a protocol of focal stroke induction in awake head-fixed mice combined with laser speckle contrast imaging blood flow assessment and mesoscopic wide-field Ca^2+^ imaging through bihemispheric transcranial windows [391]. Using transgenic GCaMP3 mice, they were able to directly observe the ischemic depolarizing wave within 5 min of stroke induction. In another series of experiments, the same group employed a similar approach to study the changes in cortical connectivity using a mouse model of small vessel disease [392]. While behavioral test results confirmed the appearance of significant motor impairments after microinfarct induction, the authors were not able to show any functional changes in neuronal activity, probably due to the insufficient resolution of this method. As a final example, Tvrdik et al. created the transgenic mouse with the GCaMP5G indicator targeted to microglia and demonstrated its applicability for studying stroke-induced Ca^2+^ waves traveling through the microglial cells in the brain [393].

The phenomenon of hypoxic pre- and post-conditioning deserves special attention. The term ischemic/hypoxic preconditioning describes a phenomenon where transient, mild hypoxic/ischemic episodes induce resistance to the damaging effects evoked by subsequent severe brain hypoxia/ischemia [394,395,396]. Ischemic/hypoxic post-conditioning is the application of transient mild hypoxic/ischemic stimuli during reperfusion after a severe hypoxic/ischemic event. Post-conditioning protects against the delayed loss of neurons after a severe hypoxic/ischemic event [397,398,399]. Remote pre- and post-conditioning are currently considered to be universal tools for the protection against hypoxic/ischemic damage of any organ, although clinical trials have produced ambiguous data regarding the utility of these approaches [400,401,402]. The effectiveness of pre- and post-conditioning for the protection against brain hypoxia/ischemia has been demonstrated in numerous studies on a variety of in vitro and in vivo experimental models [395,403]. Both pre- and post-conditioning are thought to trigger endogenous adaptive mechanisms underlying the resistance of nervous tissue to a lack of oxygen and to oxidative stress. These mechanisms remain largely unknown, although several intracellular signaling and metabolic pathways and extracellular signaling molecules have been shown to contribute to neuroprotection evoked by ischemic/hypoxic pre- or post-conditioning [395,404,405,406]. Understanding the mechanisms of neuronal resistance evoked by pre- or post-conditioning will allow researchers to identify novel molecular targets for brain hypoxia/ischemia therapies. Moreover, brain aging and many neurodegenerative diseases such as Alzheimer’s disease, Parkinson’s disease, ALS, etc., are well known to be associated with an increased oxidative stress of brain tissue [407,408,409,410]. Therefore, dissecting the mechanisms underlying neuronal resistance evoked by pre- or post-conditioning will allow for the creation of new therapeutic strategies for neuroprotection against a broad range of neurodegenerative disorders [411]. Genetically encoded reporters seem to be promising tools for the evaluation of cell signaling readjustment and cell metabolism remodeling on diverse experimental models of brain tissue protection by ischemic/hypoxic pre- and post-conditioning. For instance, monitoring signaling molecules and metabolites using genetically encoded reporters during the course of pre- or post-conditioning could reveal molecular events and biochemical processes initiating long-lasting neuroprotective effects. In the same vein, live imaging of hypoxia/ischemia on the pre-conditioned brain will allow for the identification of upregulated or downregulated cellular signaling and metabolic pathways that are essential for reducing the damaging effects of a severe hypoxic/ischemic event. Therefore, the prospect of identifying novel therapeutic targets for slowing the progression or even reversing the development of neurological deficits evoked by hypoxic/ischemic injury of the brain highlights the necessity of performing these experiments.

## 5. Conclusions

Genetically encoded reporters have great potential for elucidating the biochemical foundations of hypoxic cell damage, revealing the developmental mechanisms of ischemia-induced brain pathologies, and discovering relevant treatment methods. Such research tools are becoming increasingly popular in biomedical research. Some of these approaches allow real-time monitoring of intracellular events during hypoxia in whole living systems.

## Figures and Tables

**Table 1:** Some important characteristics of redox-sensitive fluorescent proteins (roFPs).

Name	Range Max	Midpoint Potential/EC_50_	Reference
rxYFP	2.2 (in vitro)	Midpoint potential = −261 mV	[162]
rxYFP-Grx1p	2.1 (in vitro)	Midpoint potential = −267 mV	[163]
roGFP1	~6 (in vitro)	Midpoint potential = −294 mV	[151,152]
roGFP1-Rx family	5.4–7.5	Midpoint potential −263 mV – −284 mV	[153]
roGFP1-iX	2.4–7.2	Midpoint potential −229 mV – −246 mV	[154]
roGFP2	~6 (in vitro)	Midpoint potential = −287 mV	[151,152]
roUnaG	~9 (in vitro)	Midpoint potential = −275 mV	[161]
Grx1-roGFP2	~4.4 (in living cells)	Midpoint potential = −280 mV	[155]
rxmRuby2	~2 (in vitro)	Midpoint potential = −265 ± 22 mV	[164]
rxRFP	4	Midpoint potential = −290 mV	[160]
Grx1-roCherry	1.5	Midpoint potential = −311 mV (pH = 7.0)	[158]
TrxRFP1	~6 (in vitro)	EC_50_ = 13.5 ± 0.7 μM (H_2_O_2_ in HEK293T cells) EC_50_ = 3.2 ± 1.1 μM (auranofin in HEK293T cells)	[159]

Range max—maximal dynamic range of the probe (N-fold); EC_50_—half maximal effective concentration. Background colors correspond to the color of fluorescent proteins (FPs) used as reporter domains.

**Table 2:** Some important characteristics of H_2_O_2_ and NO• probes.

Name	Range Max	Sensitivity/Kinetics	Reference
TriPer	NM	NM	[170]
HyPer	3.3 (in vitro)	Ks = 5 × 10^5^ M^−1^ s^−1^	[166,168]
HyPer-2	~6 (HeLa cells)	Ks = 1.2 × 10^5^ M^−1^ s^−1^	[167,168]
HyPer-3	~6 (HeLa cells)	Ks = 2.5 × 10^5^ M^−1^ s^−1^	[168]
HyPer7	~10 (in vitro)	i.v. = 26.9 ± 0.28 a.u./s (for HyPer3 it is 0.315 ± 0.007 a.u./s)	[173]
roGFP2-Orp1	4.8 (HeLa cells)	responds to low micromolar concentrations of exogenously applied H_2_O_2_ (HeLa cells)	[174]
roGFP2-Tsa2ΔC_R_	~6 (in vitro)	low-nanomolar or high-picomolar endogenous H_2_O_2_ concentrations	[175]
HyPerRed	~2	Ks=3 × 10^5^ M^−1^ s^−1^	[169]
geNOps group (different colors)	1.07–1.18 (HeLa cells)	EC_50_ = 50–94.1 nM	[178]

Ks—the pseudo-first order reaction rate constant, range max – maximal dynamic range of the probe (N-fold); EC_50_—half maximal effective concentration; i.v.—initial velocity (see [173]); NM—not measured. Background colors correspond to the color of FPs used as reporter domains. The last line has no background color since five probes of different colors are described in [178].

**Table 3:** Some important characteristics of NAD(H) and NADP(H) probes.

Name	Analyte	Range Max	Kd	Reference
Peredox	NADH/NAD^+^	2.5	Kd to NADH < 5 nM for initial P0 construct	[183]
Frex	NADH	9 (fl. increase)	Kd~3.7 μM at pH 7.4	[187]
FrexH	NADH	3 (fl. decrease)	Kd~ 40 nM	[187]
RexYFP	NADH/NAD^+^	~ 2 (in vitro)	K’(NADH) = 180 nM, K’(NADPH) = 6.2 μM	[184]
SoNar	NADH/NAD^+^	15	Kd(NADH) ~0.2 μM, Kd(NAD^+^) ~5.0 μM at pH 7.4	[185]
FiNad	NAD^+^/AXP	~7	Kd(NAD^+^) shifts from ~14 μM to ~1.3 mM in the presence of ATP or ADP	[189]
NAD^+^ sensor	NAD^+^	~2	Kd~65 μM	[188]
iNaps	NADPH	10	Kd = 2.0–120 μM	[186]

Kd—dissociation constant; K’—affinity constant; range max—maximal dynamic range of the probe (N-fold). Background colors correspond to the color of FPs used as reporter domains.

**Table 4:** Some important characteristics of metabolite probes.

Name	Analyte	Range Max	Kd	Reference
MaLionB	ATP	1.9	Kd = 0.46 mM	[193]
QUEEN-7μ	ATP	~6	Kd = 7.2 μM (25 °C)	[191]
QUEEN-2m	ATP	~5	Kd = 4.5 mM (25 °C)	[191]
iATPSnFR^1.0^	ATP	~3.4	EC_50_ ~ 120 μM	[192]
iATPSnFR^1.1^	ATP	~2.9	EC_50_ ~ 50 μM	[192]
mRuby-iATPSnFR^1.0^	ATP	Same as for iATPSnFR^1.0^	Same as for iATPSnFR^1.0^	[192]
MaLionG	ATP	4.9	Kd = 1.1 mM	[193]
Perceval	[ATP]:[ADP]	~2	K_R_~0.5	[197]
PercevalHR	[ATP]:[ADP]	~8	K_R_~3.5	[198]
MaLionR	ATP	4.5	Kd = 0.34 mM	[193]
ATeam1.03	ATP	~2.3	Kd = 3.3 mM (37 °C)	[194]
ATeam3.10	ATP	~2	Kd = 7.4 μM (37 °C)	[194]
GO-ATeam1	ATP	~2	Kd = 7.1 mM (37 °C)	[195]
GO-ATeam2	ATP	~3	Kd = 2.3 mM (37 °C)	[195]
BTeam (BRET)	ATP	~3	K_0.5_ = 1.7 mM (25 °C)	[196]
Laconic	Lactate	~1.2 (in vitro) ~1.4 (in living cells)	NM	[200]

Kd—dissociation constant; range max—maximal dynamic range of the probe (N-fold); EC_50_—half maximal effective concentration; K_R_—ratio of analytes at which the sensor response is half-maximal; K_0.5_—the concentration at which half of the protein molecules are bound to the analyte; NM—not measured. Background colors correspond to the color of FPs used as reporter domains. Colorless lines correspond to Förster resonance energy transfer (FRET) or bioluminescence resonance energy transfer (BRET) probes.

**Table 5:** Some important characteristics of pH probes.

Name	Range Max	pKa	Reference
ratio-pHluorin	~3 (pH 5.5–7.5)	6.9	[208]
pHluorin2	~3 (pH 5.8–7.4) in HEK293	7.1	[209,213]
EYFP	NM	7.1	[211]
E^2^GFP	~8 (pH 4.5–9.0)	~7.0	[212]
SypHer3s	>36 (pH 5.5–10.55)	7.8 in HeLa Kyoto	[172]
pHoran4	17 (pH 5.5–7.5)	7.5	[203]
pHTomato	~3 (pH 7.6–9.8)	7.8	[204]
pHuji	22 (pH 5.5–7.5 )	7.7	[203]
pHRed	>10 (pH 5.5–9.0)	6.6	[205]
mNectarine	>10 (pH 5.5–9.0)	6.9	[206]
deGFPs	NM	6.8–8.0	[210]

Range max—maximal dynamic range of the probe (N-fold); pKa—negative logarithm of acidity constant; NM—not measured. Background colors correspond to the color of FPs used as reporter domains. Colorless line corresponds to dual-emission probes.
